# Impact of the Ku Complex on HIV-1 Expression and Latency

**DOI:** 10.1371/journal.pone.0069691

**Published:** 2013-07-29

**Authors:** Gwenola Manic, Aurélie Maurin-Marlin, Fanny Laurent, Ilio Vitale, Sylvain Thierry, Olivier Delelis, Philippe Dessen, Michelle Vincendeau, Christine Leib-Mösch, Uriel Hazan, Jean-François Mouscadet, Stéphanie Bury-Moné

**Affiliations:** 1 Laboratoire de Biologie et Pharmacologie Appliquée, Centre national de la recherche scientifique-UMR8113, Ecole Normale Supérieure de Cachan, Cachan, France; 2 Regina Elena National Cancer Institute, Rome, Italy; 3 National Institute of Health, Rome, Italy; 4 Institut Gustave Roussy, Villejuif, France; 5 Institut National de la Santé et de la Recherche Médicale-U985, Villejuif, France; 6 Institute of Virology, Helmholtz Zentrum München, German Research Center for Environmental Health, Neuherberg, Germany; 7 Research Unit Cellular Signal Integration, Institute of Molecular Toxicology and Pharmacology, Helmholtz Zentrum München, German Research Center for Environmental Health, Neuherberg, Germany; 8 Department of Hematology and Oncology, Mannheim Medical Center, University of Heidelberg, Mannheim, Germany; Queensland Institute of Medical Research, Australia

## Abstract

Ku, a cellular complex required for human cell survival and involved in double strand break DNA repair and multiple other cellular processes, may modulate retroviral multiplication, although the precise mechanism through which it acts is still controversial. Recently, Ku was identified as a possible anti-human immunodeficiency virus type 1 (HIV-1) target in human cells, in two global approaches. Here we investigated the role of Ku on the HIV-1 replication cycle by analyzing the expression level of a panel of non-replicative lentiviral vectors expressing the green fluorescent protein in human colorectal carcinoma HCT 116 cells, stably or transiently depleted of Ku. We found that in this cellular model the depletion of Ku did not affect the efficiency of (pre-)integrative steps but decreased the early HIV-1 expression by acting at the transcriptional level. This negative effect was specific of the HIV-1 promoter, required the obligatory step of viral DNA integration and was reversed by transient depletion of p53. We also provided evidence on a direct binding of Ku to HIV-1 LTR in transduced cells. Ku not only promotes the early transcription from the HIV-1 promoter, but also limits the constitution of viral latency. Moreover, in the presence of a normal level of Ku, HIV-1 expression was gradually lost over time, likely due to the counter-selection of HIV-1-expressing cells. On the contrary, the reactivation of transgene expression from HIV-1 by means of trichostatin A- or tumor necrosis factor α-administration was enhanced under condition of Ku haplodepletion, suggesting a phenomenon of provirus latency. These observations plead in favor of the hypothesis that Ku has an impact on HIV-1 expression and latency at early- and mid-time after integration.

## Introduction

The human immunodeficiency virus type 1 (HIV-1) is a complex retrovirus/lentivirus bearing a genome composed of genes encoding for *i*) structural and enzymatic proteins, including the classical retroviral genes *group-specific antigen* (*gag*), *polymerase* (*pro/pol*) and *envelop* (*env*), *ii*) the regulatory proteins Transactivator of transcription (Tat) and Regulator of virion gene expression (Rev), and *iii*) the accessory proteins Viral protein R (Vpr), Viral protein U (Vpu), Virion infectivity factor (Vif) and Negative factor (Nef) [1]. The replication of HIV-1 requires two major steps: the integration of viral DNA (vDNA, *i.e.*, the provirus) into the host genome, and the expression of the integrated vDNA from its promoter, the 5' long terminal repeat (LTR) [2], [3]. This latter process is regulated by the combined action of viral and host cell proteins [3]. Briefly, the transcription of the integrated HIV-1 vDNA is initiated by the binding of ubiquitous (*e.g.*, SP1) and inducible (*e.g.*, Nuclear Factor κB, NF-κB) transcription factors of host cells to the HIV-1 promoter 5'LTR, resulting in the assembly of the cell transcription machinery and, consequently, in the production of a low amount of the viral protein Tat [3]. Tat, in turn promotes the elongation of transcription by associating with its *trans*-activation response (TAR) RNA element, a binding that allows for the recruitment of the host positive transcription elongation factor b (P-TEFb) [3].

In addition, other proteins may modulate HIV-1 expression, including Vpr, known to induce the G_2_/M arrest of HIV-1 infected cells [4], and the components of the host cell DNA repair machinery. Vpr has a stimulatory role in HIV-1 transcription [5] through a mechanism that appears to depend on the activation of the hypoxia inducible factor (HIF)-1α [6] or on the induction of the G_2_/M arrest, the cell phase in which the LTR is most active [7]. As to the DNA repair proteins, they are described to affect HIV-1 expression in either a positive (*e.g.*, DNA-dependent protein kinase (DNA-PK) [8]) or negative (*e.g.*, uracil-DNA glycosylase 2 (UNG2) [9]) fashion. In this regard, some DNA repair proteins could interact directly with HIV-1, thereby, modulating (positively or negatively) the key steps of the HIV-1 replication cycle, including the stability of vDNA (as in the case of Xeroderma Pigmentosum complementation group C/D (XPB/XPD) [10]) and the integration of the virus (*e.g.*, RAD51 [11]). As a consequence of these perturbations, HIV-1 replication in host cells may be either stimulated or inhibited. Nevertheless, the contribution of some of these DNA repair proteins remains controversial. For instance, in two previous studies performed by our group and another group, the Ku complex (also known as Ku) was reported to affect proviral gene expression in a negative [12], [13] or a positive [14] fashion, respectively.

The expression of HIV-1 may also be submitted to latency, depending on the cellular context in which infection occurs. Viral latency, which is believed to represent the major barrier against viral eradication in HIV-1-infected patients, may be established very early upon infection and leads to the generation of latent viral reservoirs [15], [16]. To date, the mechanism underlying the establishment of latency is poorly known [16]. On the contrary, either the maintenance of HIV-1 latency or the reactivation of proviruses have been extensively characterized [15], [16]. Indeed, the maintenance of HIV-1 latency has been associated to limited availability of cell transcription initiation/elongation factors, the inefficient Tat activity or some epigenetic modifications occurring on vDNA and/or nucleosomes (*e.g.*, histone deacetylation by histone deacetylases, HDACs) [16], [17]. On the other hand, the reactivation of transcriptional latency seems to involve the inhibition of HDACs and/or the activation of infected cells, which could be experimentally induced by administering to cells specific inhibitors (*e.g.*, inhibtors of HDACs (HDACi) [18]) and/or cytokines (*e.g.*, Tumor Necrosis Factor α (TNFα) [17]), respectively.

Ku is a heterodimer complex composed of two DNA-binding subunits, Ku70 and Ku80 [official gene names *x-ray cross-complementing group* (*xrcc*) *6* and *xrcc5*, respectively], displaying essential functions for human cell survival [19], [20]. Ku acts as a sensor of double strand-breaks (DSBs), thus contributing to the cellular DNA damage response [21], [22]. In particular, in the presence of DSBs, Ku recruits the kinase catalytic subunit DNA-PKcs to generate the DNA-PK complex, involved in the non-homologous end-joining (NHEJ) pathway of DSB repair [19]. Ku also plays a pivotal role in other nuclear processes, including telomere maintenance and DNA transcription (for a comprehensive description of the roles of Ku, see Ref. [23]).

Recently, two global approaches identified Ku as a potential target to limit HIV-1 replication in human cells [14], [24]. Reportedly, Ku complex may act at different stages of the HIV-1 cycle. First, we and others provided evidence that Ku contributes to the formation of 2-LTR circular circles in human and rodent cells [13], [25], [26], [27]. Moreover, Ku has been shown to directly interact with the HIV-1 integrase (IN), thereby potentially influencing the (pre-)integrative steps [25], [27], [28]. In agreement with this hypothesis, Ku is involved in provirus integration in human cells [14], [26], [27]. However, in contrast with these findings, a dispensable function of Ku and/or the DNA-PK complex in HIV-1 integration into human [29], [30], horse [30] and rodent [13], [25], [30], [31] cells has been also described. Remarkably, Ku may also be incorporated into virions during viral propagation [27], [32]. Finally, we recently showed that the Ku level may modulate provirus localization in rodent cells [13]. Nevertheless, the precise impact of Ku on both HIV-1 integration and HIV-1 expression remains unclear and requires further examinations.

To gain insights on the role of Ku in the lentiviral (LV) cycle, we performed a systematic analysis of viral expression from a collection of defective LV vectors transducing the *green fluorescent protein* (*gfp*) transgene under the control of viral or cellular promoters on wild-type (WT) and *Ku80* ^+/−^ human colorectal carcinoma HCT 116 cells. With this strategy, we demonstrated that Ku plays a dual function in response to HIV-1 infection, namely to promote the early transcription from the HIV-1 promoter and to limit the constitution of viral latency. Ku depletion had no impact on the (pre-)integrative steps, but rather decreased HIV-1 expression by acting at the transcriptional level. Consistently, Ku associates to HIV-1 LTR in transduced cells. Also, we put in evidence that that the knockdown of p53 in Ku80-haplodepleted cells was accompanied by both increased Ku protein levels and enhanced HIV-1 expression. Finally, we showed that the depletion of Ku limited the counter-selection of HIV-1-expressing cells over time and favored the establishment of cells containing latent viruses, which may be readily reactivated by the administration of the HDACi trichostatin A (TSA) or the NF-κB activator TNFα.

## Results

### The Level of Ku Influences the Efficiency of HIV-1-driven Expression

To investigate the effects of Ku on the HIV-1 replication cycle, we took advantage of heterozygous *Ku80^+/−^* human colon carcinoma HCT 116 cells [33]. These cells represent a valid model for Ku haplodepletion for at least three reasons: (*i*) they are characterized by an expression of Ku80 (at both the mRNA and protein levels) that is half that of parental *Ku80^+/+^* (WT) cells ( [33] and **Figure S1A,B**), (*ii*) as the binding to Ku80 stabilizes Ku70, they also manifest a marked (approximately 50%) depletion of Ku70 ([33] and **Figure S1B**), (*iii*) they display an elevated sensitivity to ionizing radiation for the decrease in Ku activity [33], and (*iv*) they present similar proliferation rate and comparable cell cycle profiling of their WT counterparts (**Figure S1C**).

**Figure 1:**
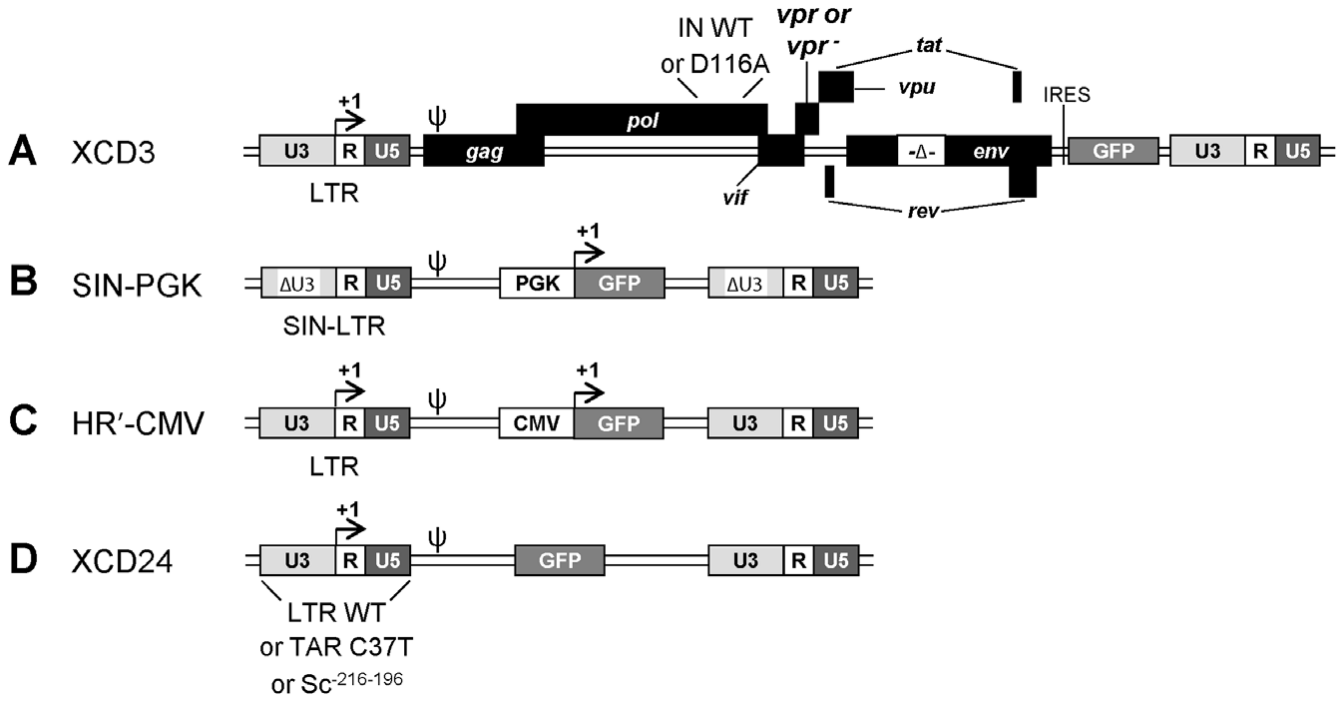
Design of lentiviral vectors. (**A**) Schematic representation of the lentiviral (LV) vectors (in the proviral, integrated form) used in this study. XCD3 is a human immunodeficiency virus type 1 (HIV-1) construct lacking the *envelop* (*env*) and *negative factor* (*nef*) genes and carrying the *green fluorescent protein* (*gfp*) transgene under the control of the native HIV-1 long terminal repeat (LTR) and an internal ribosome entry site (IRES) (also referred as to HIV-1 *env^-^ nef ^-^* IRES-*gfp*). Two derived versions of this vector have been engineered, the first presenting the deletion of Vpr (*vpr^-^*) and the second the D116A mutation in the integrase (IN) coding sequence [named XCD3 *vpr^-^* and XCD3 IN(D116A), respectively]. (**B**) SIN-PGK is a self-inactivating (SIN, *i.e.*, U3-deleted, ΔU3) LV vector containing the *gfp* under the control of the human phosphoglycerate kinase (PGK) internal promoter. (**C**) HR’-CMV is an LV vector carrying the *gfp* transgene under the control of both the immediate early human cytomegalovirus (CMV) and HIV-1 promoters. (**D**) XCD24 is an LV vector lacking all HIV-1 genes and carrying the *gfp* under the control of the wild-type (WT) HIV-1 promoter. Two variants of this vector have been produced, the first mutated in the putative Ku binding site [KBS, XCD24 Sc^-216-196^] and the second in the *trans*-activation response (TAR) element [XCD24 TAR(C37T)]. ψ, encapsidation signal.

**Figure 2:**
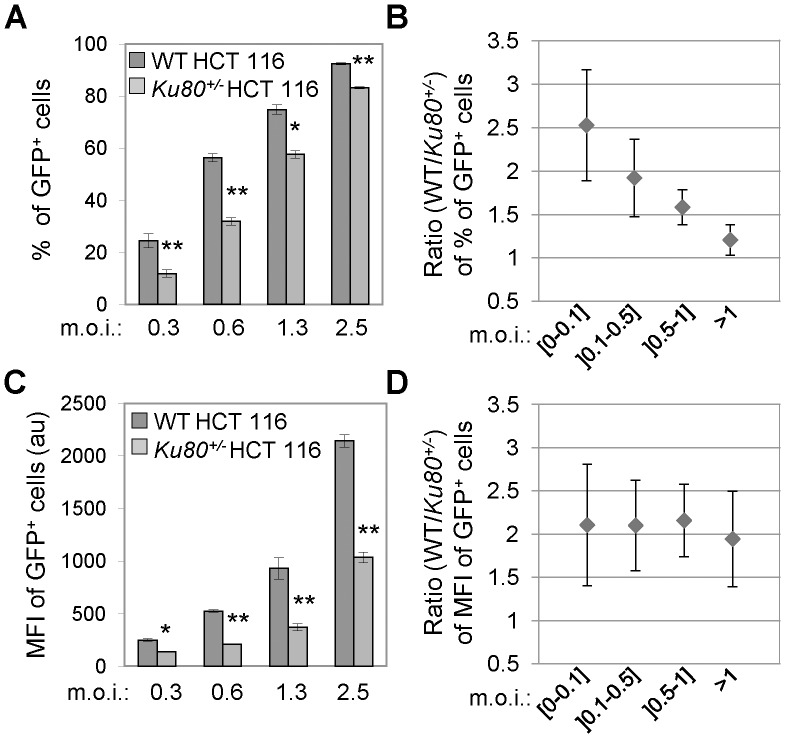
Ku80 haplodepletion reduces HIV-1-driven GFP expression. (**A**–**D**) Wild-type (WT) and *Ku80^+/−^* human colon carcinoma HCT 116 cells were transduced at the depicted multiplicity of infection (m.o.i.) with XCD3 (HIV-1 *env^-^ nef ^-^* IRES-*gfp*) as indicated and were analyzed 48 h later by cytofluorometry for the percentage and the geometric mean fluorescence intensity (MFI) of green fluorescent protein-positive (GFP^+^) cells. Panels (**A**) and (**C**) present the results of the percentage and the MFI of GFP^+^ cells, respectively, obtained from at least two independent experimentations at the indicated m.o.i. (mean ± SD; *, p<0.05; **, p<0.01), except for the last m.o.i. for which one representative experiment (of 2 independent observations yielding similar results) is presented (mean ± SD). The ratios between WT and *Ku80^+/−^* HCT 116 cells for the percentage and the MFI of GFP^+^ cells at the depicted m.o.i. are reported in panels (**B**) and (**D**), respectively (mean ± SD, n  = 3). au, arbitrary units.

We first evaluated the effect of Ku depletion on HIV-1 transduction efficiency. To this end, WT and *Ku80^+/−^* HCT 116 cells were transduced with XCD3 - an *env*-defective HIV-1 construct in which *nef* is replaced by a *gfp* transgene under the control of the native HIV-1 LTR and an internal ribosome binding site (IRES) (**Figure 1A**) - followed by the cytofluorometry-mediated assessment of GFP expression. When performing this analysis at a low multiplicity of infection (m.o.i. of 0.3), we observed that the percentage of GFP-positive (GFP^+^) cells among *Ku80^+/−^* HCT 116 cells was approximately half that of their WT counterparts (**Figure 2A,B**). Moreover, as compared to *Ku80^+/+^* cells, transduced *Ku80^+/−^* HCT 116 cells displayed lower GFP expression levels, as monitored by the geometric mean fluorescence intensity (MFI) (**Figure 2C,D**). At high m.o.i., the percentage of GFP^+^ cells among the Ku80-haploinsufficient population was similar in value to that observed among WT cells, and this is likely due to saturation of the number of cells expressing the transgene (**Figure 2A,B**). However, the difference in MFI of GFP^+^ cells was still conserved (**Figure 2C,D**), indicating that Ku depletion affects transgene expression even at high m.o.i. XCD3 transduction had no significant effect on proliferation/viability in either WT or *Ku80^+/−^* HCT 116 cells, as evaluated by a colorimetric assay performed 48 h post-transduction (data not shown), thus excluding a potential loss of transduced cells.

**Figure 3:**
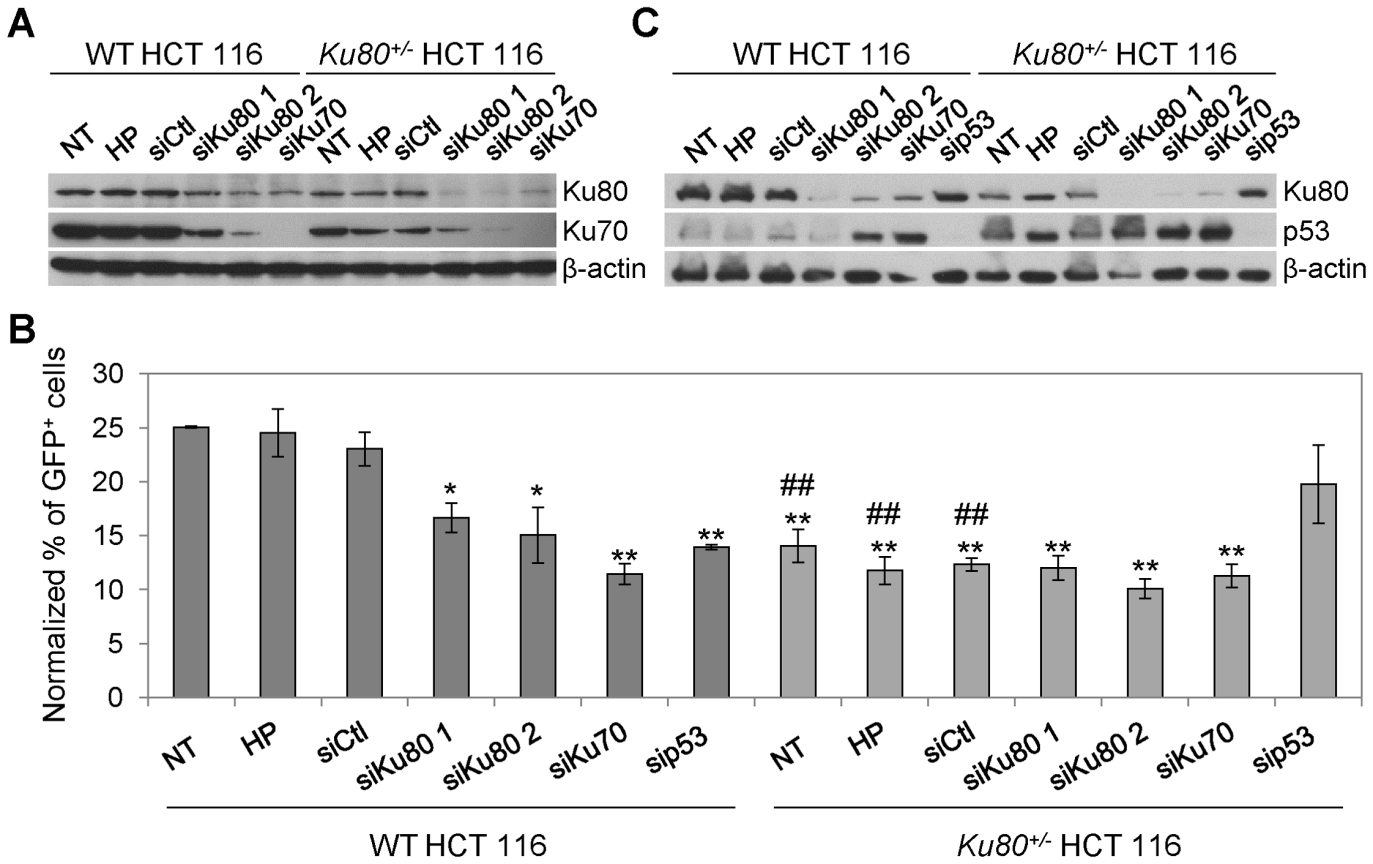
HIV-1-driven GFP expression in WT HCT 116 cells is decreased by transient depletion of Ku. (**A**–**C**) Wild-type (WT) and *Ku80^+/−^* human colon carcinoma HCT 116 cells were transfected with an unrelated sequence (siCtl) or with siRNAs directed against Ku80 (siKu80 1 or 2), Ku70 (siKu70) or p53 (sip53). Seventy-two hours later, the protein levels of Ku80 and Ku70 (**A**) or Ku80 and p53 (**C**) were assessed by Western-blot analysis. Actin levels were monitored to ensure equal loading of lanes. Alternatively, 72 h upon transfection, the cells were transduced with XCD3 (HIV-1 *env^-^ nef ^-^* IRES-*gfp*) at low m.o.i. (<0.3) for additional 48 h followed by cytofluorimetric assessment of green fluorescent protein (GFP) expression (**B**). NT (non-transfected) and HP (Hiperfect®) correspond to cells kept in control condition or exposed to the same amount of liposomes as used for transfection but in the absence of siRNA, respectively. In (**B**), the percentages of GFP-positive (GFP^+^) cells were normalized to the total viral DNA content per cell as determined by quantitative PCR (Q-PCR) 24 h after transduction. The results obtained from at least 3 independent experiments were then normalized to a GFP expression level of 25% (of note, the level of GFP expression of NT, HP or siCtl WT cells before normalization was always comprised between 20 and 30%). The results are expressed as mean ± SEM. *, p<0.05 and **, p<0.01 as compared to HP and siCtl-transfected WT cells; ^#^, p<0.05 and ^##^, p<0.01 as compared to WT cells subjected to the same transfection condition.

To confirm these results, we performed additional experiments in which WT and *Ku80^+/−^* HCT 116 cells were transiently depleted of Ku by means of transfection with small interfering (si) RNAs directed against either Ku80 or Ku70 (**Figure 3A**). Seventy-two hours after transfection, the cells were transduced with XCD3 for additional 48 h, and then analyzed by cytofluorometry for transgene expression. As shown in **Figure 3B**, the knockdown of Ku significantly decreased HIV-1 expression levels in WT cells. On the contrary, in *Ku80^+/−^* HCT 116 cells, the transgene expression was not altered by the small interfering (si) RNAs further depleting Ku (**Figure 3B**), suggesting that a ∼50% depletion of Ku is already sufficient to affect HIV-1 expression.

Taken together, these observations demonstrate that either the prolonged (**Figure 2**) or the transient (**Figure 3**) depletion of Ku in target cells negatively affects GFP expression from the HIV-1 promoter.

### Ku and p53 may Cooperate to Modulate HIV-1 Expression

In accordance with data previously reported by others [33], [34], we observed that the basal level of p53 was higher in Ku80-haploinsufficient cells than in their WT counterparts (**Figures 3C, S** **1D**). Intrigued by this finding, we analyzed in depth the impact of p53 on Ku80 expression and, consequently, on transgene expression. We found that both the knockdown (by transfecting specific p53-depleting siRNA, **Figure 3C**) and the knockout of p53 (by using *TP53^−/−^* HCT116 cells, **Figure S2**) were associated with an increase in the amounts of both Ku70 (not shown) and Ku80 (**Figures 3C, S** **2A**). Intriguingly, the expression of the transgene from XCD3 was subjected to a decrease in WT HCT 116 cells by p53 depletion (**Figure 3B**). Along similar lines, *TP53^−/−^* HCT 116 cells displayed a lower HIV-1 expression in comparison to WT cells (**Figure S2B**). On the contrary, XCD3 expression was increased in *Ku80^+/−^* HCT 116 cells depleted of p53, reaching a level that was intermediate between those of untransfected WT and *Ku80^+/−^* HCT 116 cells (**Figure 3B**). Although a direct role for p53 on HIV-1 transduction/expression cannot be excluded, this latter finding could be ascribed to the increased Ku protein levels in *Ku80^+/−^* cells as provoked by the depletion of p53.

To confirm this hypothesis, we performed experiments in which we transiently or stably *trans*-complemented Ku in *Ku80^+/−^* HCT 116 cells by means of Ku80-expressing plasmids or viral vectors (either alone or combined with the fluorescent protein DsRed). Thereafter, the polyclonal cell population as well as multiple DsRed^+^ cell clones isolated by cytofluorometry-assisted cell sorting were assessed for Ku protein levels. In addition, we took advantage of a commercially available Ku *trans*-complemented *Ku80^+/−^* HCT 116 clone (named pKu80 *Ku80^+/−^* clone). In none of these experiments, which were repeated at least twice yielding similar results, we observed an increase in Ku expression upon *trans*-complementation (data not shown and **Figure S3** for pKu80 *Ku80^+/−^* clone), a finding possibly highlighting the intrinsic difficulty in modifying the expression of Ku in a stable fashion in these p53-proficient cells.

In conclusion, we provide experimental evidence on a process tightly linking the expression levels of Ku and p53 that appears relevant for HIV-1 expression.

### Impact of Ku Level on the (pre-)integrative Steps of the HIV-1 Cycle

To elucidate the impact of the Ku level of target cells on the pre-integrative steps of the HIV-1 replication cycle, we determined by quantitative PCR (Q-PCR) all vDNA species - namely, total vDNA, 2-LTR circular viral forms, and integrated vDNA - in WT and *Ku80^+/−^* HCT 116 and in T lymphoid leukemia (Jurkat and CEM-T4) cells previously transduced with XCD3.

**Figure 4:**
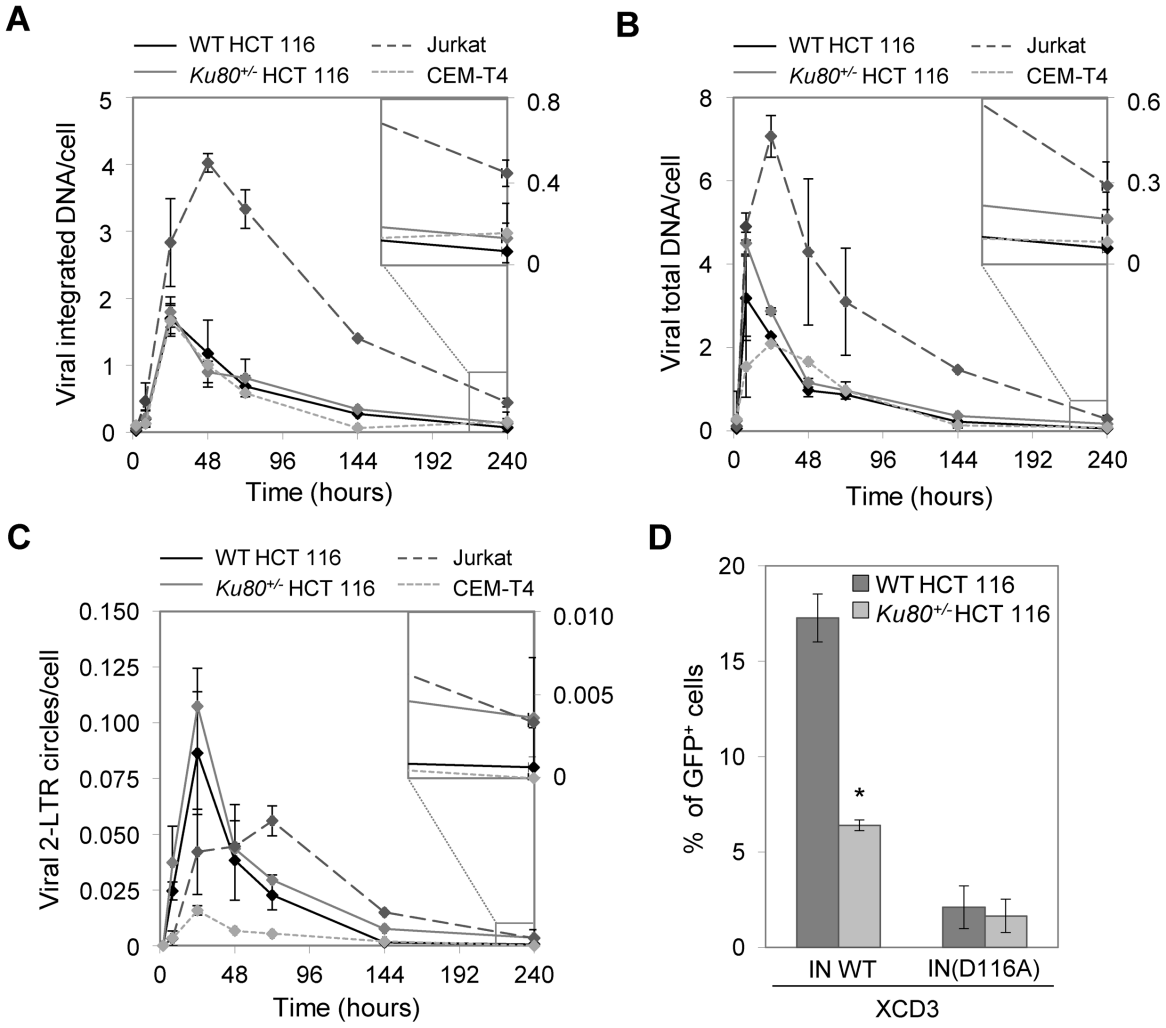
The haplodepletion of Ku in target cells has no effect on HIV-1 (pre-)integrative steps but requires viral integration to modulate HIV-1 expression. (**A–C**) Wild-type (WT) and *Ku80^+/−^* human colon carcinoma HCT 116 cells as well as the human T lymphoid leukemia Jurkat and CEM-T4 cell lines were transduced with the same volume of XCD3 (m.o.i <0.2). At the time specified from transduction, the cells were subjected to quantitative PCR (Q-PCR) assays to quantify the viral DNA (vDNA) species. The copy number of the integrated vDNA (**A**), the total vDNA (**B**) and the 2-long terminal repeat (LTR) circular forms of vDNA (**C**) per cell for each cell line are reported (mean ± SEM; n  = 2). The last data point (240 h post-transduction) is shown at higher magnification. (**D**) WT and *Ku80^+/−^* HCT 116 cells were transduced for 48 h either with XCD3 or its integrase-defective derivative, XCD3 IN(D116A) (at a m.o.i.  = 0.15), followed by cytofluorometry-mediated assessment of the percentage of green fluorescent protein-positive (GFP^+^) cells. One representative experiment of at least 2 independent observations yielding similar results is represented (mean ± SD; *, p<0.05).

Of note, as determined in parallel by cytofluorometric assessments, the kinetic of GFP expression was comparable in WT HCT 116, Jurkat and CEM-T4 cells, and was in all cases higher than in *Ku80^+/−^* HCT 116 cells (**Figure S4**). As demonstrated by Q-PCR analyses, XCD3 was efficiently integrated into the genome of both *Ku80^+/+^* and *Ku80^+/−^* HCT 116 cells. Indeed, ∼50% of total vDNA was integrated into the cellular genome 24 h post-transduction (**Figure 4A**), a percentage that is consistent with previous reports [35]. Similar levels of vDNA integration were also observed in both CEM-T4 and Jurkat cells (**Figure 4A**). Moreover, in our hands, the decrease in integrated or total vDNA was more rapid in HCT 116 and CEM-T4 cells than in Jurkat cells (**Figure 4A,B**). Importantly, no difference in the quantity of integrated vDNA and of vDNA present as 2-LTR circular forms was observed between *Ku80^+/+^* and *Ku80^+/−^* HCT 116 cells at early times (2 to 72 h) following XCD3 transduction (**Figure 4A,C**). Nevertheless, at increased times (10 days post-transduction), the levels of total and integrated vDNA per cell were higher in *Ku80^+/−^* HCT 116 cells than in their WT counterparts (**Figure 4A,B**).

These results demonstrate that depletion of Ku in transduced HCT 116 cells does not affect the capacity of the vDNA to form 2-LTR circles or to become integrated into the cellular genome.

Given that Ku may be incorporated into virions [27], [32] and that this incorporation may positively affect viral expression [27], we performed experiments in which XCD3 or self-inactivating (SIN)-PGK vectors [which contains the human phosphoglycerate kinase (PGK) internal promoter, **Figure 1B**] were produced in human embryonic kidney (HEK) 293T cells previously depleted of Ku (by means of 24 h transfection with either Ku80- or Ku70-depleting siRNAs) (**Figure S5A**,**B**). Upon their production, the vectors were transduced in WT and *Ku80^+/−^* HCT 116 target cells, followed by Q-PCR-mediated analysis of vDNA species or by cytofluorometry-mediated assessment of GFP expression 24 or 48 h later, respectively (**Figure S5A–F**). In these experiments, we observed that the depletion of Ku in virus producing HEK293T cells decreased the quantity of total vDNA (normalized to the amount of the viral protein p24 per cell) of - and thus the efficiency of retrotranscription from - XCD3 or SIN-PGK vectors in both WT (**Figure S5C**) and *Ku80^+/−^* (data not shown) cells, without interfering with the capacity of the vectors to either form 2-LTR circles or integrate into host genomes (data not shown). Accordingly, also viral expression (normalized to the amount of p24 per cell) from vectors containing the HIV-1 LTR (*i.e.*, XCD3) or the internal promoter PGK (*i.e.*, SIN-PGK) was reduced in both WT and *Ku80^+/−^* cells when virus particles were produced in Ku-deficient cells (**Figure S5D–F**). Nevertheless, depletion of Ku during viral particle production did not modify the difference in GFP expression level between WT and *Ku80^+/−^* cells (**Figure S5E**,**F**).

These results confirm that encapsidation of Ku into viral particles has a positive impact on viral transduction, and demonstrate for the first time that this effect is independent on either HIV-1 accessory proteins (that are not encoded by the packaging plasmid used to produce SIN-PGK vectors), the promoter contained in the vector, or the Ku level of target cells.

Thereafter, we investigated whether Ku would exert a similar effect on the expression of human endogenous retroviruses (HERV). To this aim, we performed a comprehensive microarray-based analysis of the transcriptional activity of HERVs in WT and *Ku80^+/−^* HCT 116 cells (**Figure S6**), followed by reverse transcription (RT-) Q-PCR experiments (**Table S1**). No significant difference was observed between WT and *Ku80^+/−^* HCT 116 cells, thus confirming that the impact of Ku is specific for HIV-1 expression.

To investigate whether Ku modulation of HIV-1 expression requires viral integration, we compared the GFP expression after transduction with a vector containing the HIV-1 promoter efficient (XCD3) or deficient for integration, *i.e.*, harboring the IN D116A mutation [XCD3 IN(D116A)] (**Figure 1A**). We observed no effect of Ku on the basal levels of transgene expression from unintegrated vDNA forms in cells transduced with XCD3 harboring the IN D116A mutation (**Figure 4D**), suggesting that the Ku complex requires integration to impact on HIV-1 expression.

In conclusion, the Ku level of target cells has no impact on the (pre-)integrative forms of the HIV-1 replication cycle, but specifically promotes the expression of the integrated form of HIV-1 genome.

### Ku Depletion Negatively Affects the HIV-1 Expression via a Transcriptional Mechanism

Given that Ku has been implicated in the transcriptional regulation of various genes (*e.g.*, human *interleukine 2* (*IL2*) [36], human *xanthine oxidoreductase* [37], mouse mammary tumor virus (MMTV) [38], [39]), we wondered whether this complex also controls transcription from the HIV-1 promoter, which directs transgene expression.

**Figure 5:**
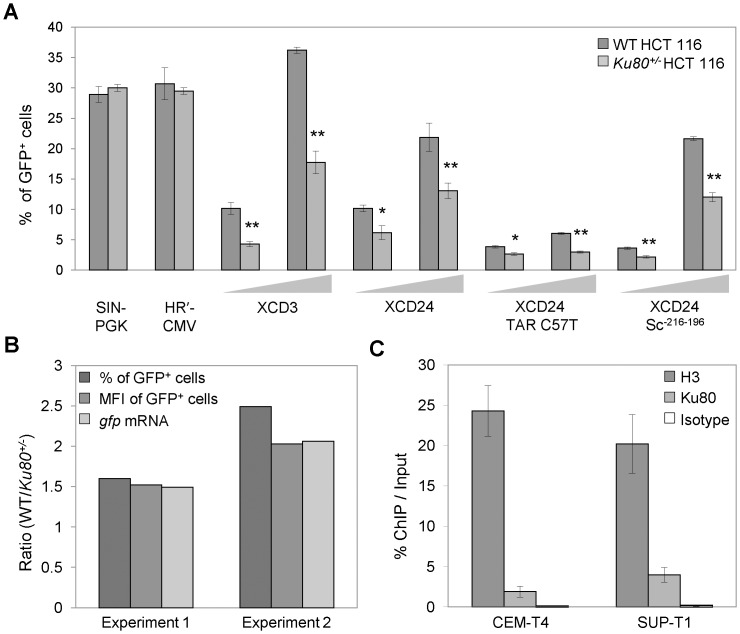
The level of Ku impacts specifically on the LTR-driven GFP transcription from HIV-1. (**A**) Wild-type (WT) and *Ku80^+/−^* human colon carcinoma HCT 116 cells were transduced for 48 h at the depicted m.o.i. either with SIN-PGK, HR’-CMV, XCD3, XCD24 or derivatives of XCD24 in which the putative Ku-binding sequence (KBS) (XCD24 Sc^-216-196^) or the *trans*-activation response (TAR) RNA element [XCD24 TAR(C37T)] was mutated (for more details on vectors, refer to **Figure 1**). Thereafter, the percentage of green fluorescent protein-positive (GFP^+^) cells was measured by flow cytometry. The results from at least 2 independent experiments are presented as mean ± SEM (*, p<0.05; **, p<0.01), except for XCD24 Sc^-216-196^ and XCD24 TAR(C37T) for which one representative experiment (of 2 independent observations yielding similar results) is represented (mean ± SD). (**B**) WT and *Ku80^+/−^* HCT 116 cells were transduced at mid m.o.i. (<0.5) with XCD3 and subjected 48 h later to either flow cytometric analyses to measure the percentage and the geometric mean fluorescence intensity (MFI) of GFP^+^ cells or reverse transcription quantitative PCR (RT-Q-PCR) assays to quantify the *gfp* mRNA content (normalized to *gapdh* mRNA content). The ratios between WT and *Ku80^+/−^* cells HCT 116 cells for the indicated parameters are illustrated. The results correspond to two representative and independent experiments. Of note, the percentages of GFP^+^ WT cells in experiments 1 and 2 were of 15% and 25%, respectively. (**C**) Human T lymphoid leukemia (CEM-T4) and lymphoma (SUP-T1) cells were transduced for 48 h with human immunodeficiency virus type 1 (HIV-1) NL4.3 *env* ^-^ before chromatin immunoprecipitation (ChIP)-mediated assessment of the binding of Ku80 to the region of the HIV-1 long terminal repeat (LTR) and the *leader* region, which is located downstream of the LTR. The results are expressed as the fraction of immunoprecipitated DNA for each set of conditions (mean ± SEM, n  = 2). H3, Ku and isotype correspond to conditions in which the DNA was immunoprecipitated by using specific antibodies against histone H3 or Ku80, or an irrelevant control antibody, respectively.

For this, we conducted transduction experiments with LV vectors in which *gfp* was placed under the control of the PGK (*i.e.*, SIN-PGK vector, **Figure 1B**) or the cytomegalovirus immediate early (CMV) internal promoter (*i.e.*, the HR’-CMV vector, **Figure 1C**). As opposed to what was observed for XCD3, the percentage of GFP^+^ cells after transduction with SIN-PGK or HR’-CMV was comparable in WT and *Ku80^+/−^* HCT 116 cell lines (**Figure** **5A**). Thus, the level of Ku in target cells appears to have no significant impact on the GFP expression from an internal heterologous promoter (*e.g.*, PGK or CMV), pointing to a specific role of Ku on the HIV-1 promoter.

We then evaluated the impact of HIV-1 regulatory and accessory proteins on this phenomenon. To this aim, we took advantage of XCD24, a vector containing *gfp* gene under the control of the HIV-1 promoter and deleted of all HIV-1 coding sequences (**Figure 1D**). Consistent with our observations on XCD3, GFP expression from XCD24 was reduced in Ku-depleted cells as compared to their WT counterparts, and this at different m.o.i. (**Figure 5A**). Similar results were obtained with an XCD3 variant lacking Vpr (**Figure S7A**,**B**), *de facto* excluding that HIV-1 regulatory and accessory proteins are required for the effects of Ku on HIV-1 expression.

To rule out the possible contribution of Tat proteins coming from virus producing cells, we employed a XCD24 variant carrying the C37T mutation in the TAR element [XCD24 TAR(C37T), **Figure 1D**], which affects Tat responsiveness [40]. Both WT and *Ku80^+/−^* HCT 116 cells were less efficiently transduced by this vector, as compared to XCD3 (**Figure 5A**), owing to defective virus production (for more details, refer to [40]). Nonetheless, this decrease was more pronounced in *Ku80^+/−^* cells than in their WT counterparts, confirming that the effect of Ku on the expression of the HIV-1-based constructs is independent from Tat. Similar results were also recorded with the XCD24 Sc^-216-196^ vector, carrying a scrambled sequence in place of a previously identified Ku-binding sequence (KBS), extending from nucleotides -216 to -196 in the U3 LTR [12] (**Figure 1D**), suggesting that Ku influences transgene expression without binding to the HIV-1 putative KBS^-216-196^ sequence (**Figure 5A**).

Thereafter, we explored the impact of Ku depletion on HIV-1 transcription. For this, WT and *Ku80^+/−^* HCT 116 cells were transduced with XCD3 followed by RT-Q-PCR-mediated assessment of *gfp* RNAs 48 h later. Lower levels of GFP-encoding mRNAs were detected in *Ku80^+/−^* HCT 116 cells 2 days after transduction, than in their WT counterparts (**Figure 5B**). Indeed, the quantitative difference between GFP-encoding mRNAs detected in *Ku80^+/+^ vs. Ku80^+/−^* cells correlated with that observed in GFP expression levels (**Figure 5B**), suggesting that Ku influences HIV-1 expression by acting at the transcriptional level.

Finally, we performed chromatin immunoprecipitation (ChIP) analyses on T lymphoid lymphoma (SUP-T1) and leukemia (CEM-T4) cells - two cell lines physiologically more relevant for HIV-1 infection, but less easily transfectable with, and thus depletable of, Ku as compared to those used throughout this article - previously transduced for 48 h with HIV-1 NL4.3 *env* ^-^ aimed at assessing the binding of Ku80 to a site that includes a portion of the HIV-1 LTR (a part of the U3 region plus the complete R and U5 regions) and the *leader* region, which is located downstream of the LTR (**Figure 5C**). Noteworthy, this site is recognized by several cell transcription initiation and elongation factors (including activator protein 1 (AP-1), interferon regulatory factor (IRF), NF-κB, nuclear factor of activated t-cells (NFAT), SP1 [17], [41], [42], [43], [44]). As shown in **Figure 5C**, upon viral transduction, Ku80 is able to associate with 2% (in CEM-T4) or 4% (in SUP-T1) of vDNA.

Taken together, these observations demonstrate that the depletion of Ku negatively affects GFP transcription of HIV-1, suggesting that Ku enhances basal HIV-1 transcription through a direct binding to the HIV-1 LTR.

### The Reactivation of Latent HIV-1 is Favored in Ku80-haplodepleted Cells

**Figure 6:**
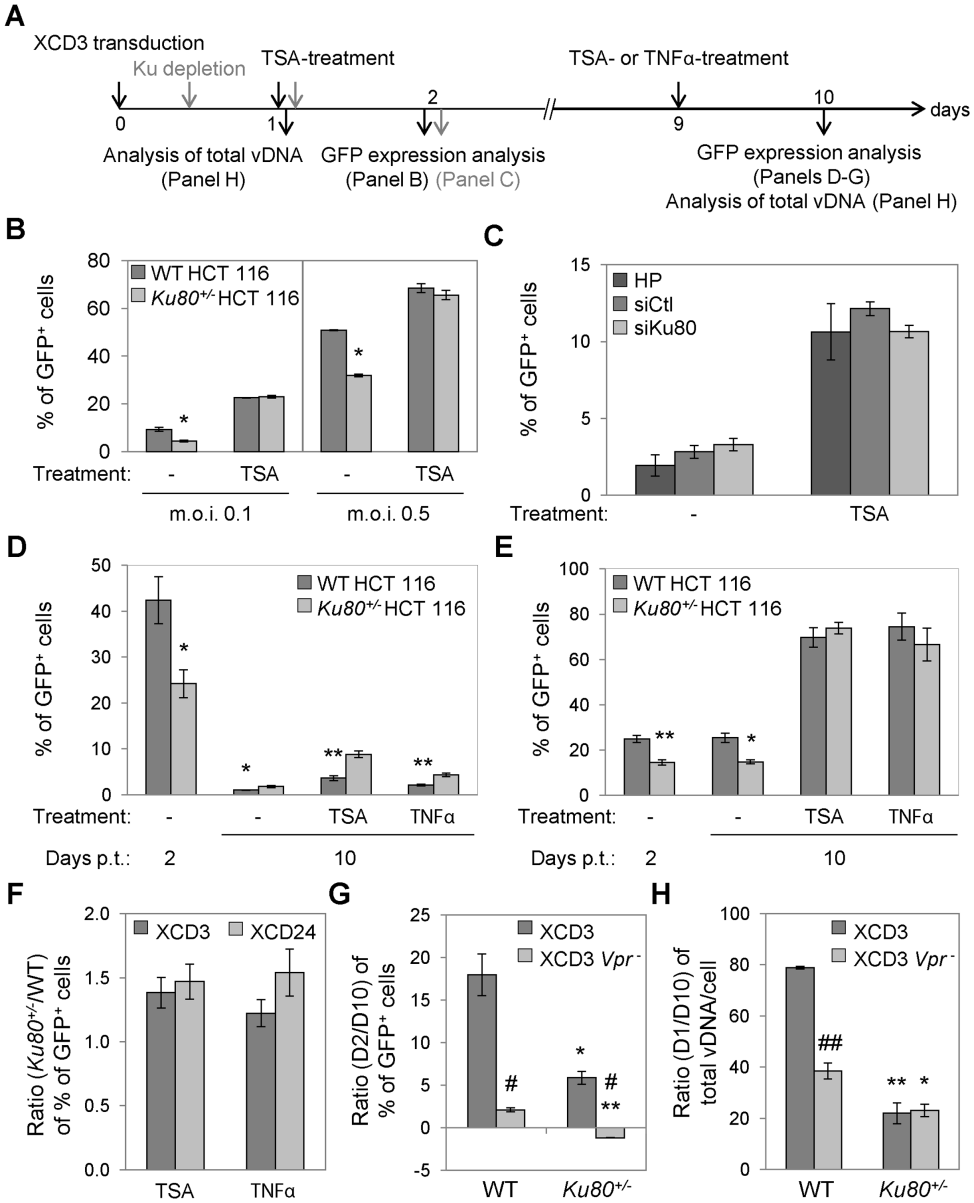
Ku80 haplodepletion increases the level of TNFα- or TSA-induced HIV-1 reactivation. (**A**) Schematic representation of the experimental strategy adopted in this figure. (**B–D**) Wild-type (WT) and *Ku80^+/−^* human colon carcinoma HCT 116 cells transduced with XCD3 (at an m.o.i. <0.6) (**B**,**D**), and WT HCT 116 cells transfected with an unrelated sequence (siCtl) or with siRNAs directed against Ku80 (siKu80) 8 h after XCD3 transduction (**C**), were incubated for 24 h in the absence or presence of trichostatin A (TSA) or tumor necrosis factor α (TNFα) either 24 h (**B**,**C**), or 9 days upon transduction (**B**,**D**). The percentage of green fluorescent protein-positive (GFP^+^) cells was then estimated by flow cytometry. In panel (**C**), HP (Hiperfect®) corresponds to cells transfected only with the same amount of liposomes used for siRNA transfection. In panel (**B**), the results of two independent experimentations performed in duplicate are reported (mean ± SD, n  = 4). *, p<0.05, as compared to WT cells. Panels (**C**,**D**) show results from 2 and 4 independent experiments, respectively (mean ± SEM). *, p<0.05; **, p<0.01, as compared to WT cells. (**E**) WT and *Ku80^+/−^* HCT 116 cells transduced with XCD24 were treated 9 days later with TSA or TNFα as described in (**D**) (mean ± SEM, n  = 3). *, p<0.05; **, p<0.01, as compared to WT cells. (**F**) Ratio between the percentage of GFP^+^ cells of *Ku80^+/−^* and WT cells in the presence or absence of TSA or TNFα, as determined in (**D**) and (**E**) for the depicted lentiviral (LV) vectors (mean ± SD, n  = 3). (**G**) Ratio between the percentage of GFP^+^ cells observed upon 2 or 10 days from transduction, as determined in (**D**) for XCD3 and in (**Figure S7A**) for XCD3 *vpr^-^* on WT and *Ku80^+/−^* cells (mean ± SEM, n  = 2). *, p<0.05; **, p<0.01, as compared to WT cells; ^#^, p<0.05, as compared to XCD3-transduced cells. (**H**) Ratio between the quantity of viral DNA (vDNA) 1 or 10 days upon transduction, as determined in (**D**) for XCD3 and (**Figure S7C**) for XCD3 *vpr^-^*, on WT and *Ku80^+/−^* cells (mean ± SEM, n  = 2). *, p<0.05; **, p<0.01, as compared to WT cells; ^##^, p<0.01, as compared to XCD3-transduced cells. p.t., post-transduction.

Finally, we analyzed the role of Ku in the events that are associated with the establishment/maintenance of provirus latency and with the reactivation of latent proviruses. To address this issue, transgene expression was evaluated in WT and *Ku80^+/−^* HCT 116 cells 2 or 10 days upon transduction with XCD3 followed by exposure to either the TNFα (which promotes HIV-1 LTR-driven transcription by activating the transcription factor NF-κB [45]), or the HDACi TSA (which stimulates transgene expression by promoting histone acetylation [46]) (**Figure 6A**). Of note, the use of TNFα and TSA is a standard experimental approach for studying the reactivation from viral latency [47], [48].

When administering TSA 24 h upon XCD3 transduction, transgene expression were stimulated in both WT and *Ku80^+/−^* HCT 116 cells, while the difference in GFP expression between these cell lines observed in the absence of TSA was abolished, thereby revealing the existence of a pool of TSA-responsive GFP^-^ cells in the transduced *Ku80^+/−^* population (**Figure 6A,B**). To further study the impact of Ku in the establishment/maintenance of this pool, WT HCT 116 cells were depleted of Ku (by means of Ku80-depleting siRNA transfection, **Figure S8A**) 8 h upon transduction with XCD3, followed by exposition or not to TSA 24 h later. As expected, TSA administration enhanced transgene expression irrespectively to the level of Ku (**Figures 6A,C**). Nevertheless, as opposed to what was previously observed (**Figure 6B**), the depletion of Ku after transduction did not induce significant modifications in the level of GFP expression from XCD3 either in the absence or presence of TSA, and this after 48 h (**Figures 6C**) and 72 h (**Figure S8B**) from transduction. These observations not only confirm the role of Ku in inducing HIV-1 expression, but also suggest that Ku level may be crucial for the entry in and/or the maintenance of latency of HIV-1 during the early phases upon transduction. In line with this finding, the increase in GFP expression induced by 24 h of TNFα- or TSA-treatment 9 days post-transduction with XCD3 was higher in *Ku80^+/−^* than in WT cells (**Figures 6A,D**).

To further evaluate the mechanisms through which the depletion of Ku contributes to the establishment/maintenance of or the reactivation from HIV-1 latency, we took advantage of the Tat-deficient vector XCD24 - a condition that reportedly induces virus pseudo-latency [49] - or the Vpr-deficient vector XCD3 *vpr* ^-^. The (re-)activation of transgene expression by TNFα or TSA was observed in WT and *Ku80^+/−^* HCT 116 cells transduced 9 days earlier with XCD24 (**Figure 6E**). Similarly to what was reported for XCD3, the ratio between the percentage of GFP^+^ cells in the presence or absence of TSA or TNFα was higher in Ku80-haplodepleted than in WT cells (**Figure 6F**). These observations suggest that a pool of TSA-responsive GFP^-^ cells is present at early and mid times upon XCD3 transduction in both cell lines and that this fraction of cells harboring a silent provirus in a reactivatable state is higher in conditions of Ku-depletion. In addition, as opposed to what was observed with XCD3, WT and *Ku80^+/−^* cells transduced with XCD24 (**Figure 6E**) or with the XCD3 *vpr* ^-^ vector (**Figures 6G, S** **7C**) failed to exhibit or presented a limited decrease in transgene expression over time, respectively. In line with this result, a higher amount of vDNA was observed in WT HCT 116 cells with XCD3 *vpr* ^-^ than in those transduced with XCD3 10 days after transduction (**Figure** **6H**). Of note, XCD3 *vpr* ^–^transduced WT cells displayed a lower vDNA quantity than XCD3 *vpr* ^–^transduced Ku80-haplodepleted cells upon 10 days from transduction (**Figure** **6H**).

These results suggest that the HIV-1 accessory proteins, including Vpr, may promote the counter-selection/elimination of cells expressing the integrated virus, and this especially in WT cells that have a higher level of HIV-1 transcription.

As expected, the administration of reactivating agents did not modify transgene expression from SIN-PGK in both WT and Ku80-haplodeficient cells (data not shown). Of note, the percentage of GFP expression from this latter vector was stable over time (data not shown). Finally, Ku level altered neither the kinetic of transgene extinction nor the percentage of GFP reactivation in GFP^+^ HCT 116 cells isolated 2 days post-transduction by cytofluorometry and incubated or not for 24 h with TSA 2 or 4 weeks later (**Figure S9**).

Altogether, these results suggest that the depletion of Ku in target cells may favor the early establishment and/or maintenance of a pool of TSA-responsive GFP^-^ cells bearing an integrated, latent and readily (re-)activatable provirus, and being less subjected to counter-selection over time.

## Discussion

Here, we investigated the impact of Ku level on LV replication cycle by transducing distinct non-replicative LV vectors encoding the *gfp* transgene.

First, we demonstrated that the depletion of Ku in target cells does not affect the (pre-)integrative steps of the HIV-1 replication cycle. These results are in apparent contradiction with some previous observations made by our group and other groups, which described a positive role of Ku in the formation of 2-LTR circular circles [13], [25], [26], [27]. As detailed in the introduction, the impact of Ku complex on provirus integration is still debated, and this is likely due to the intrinsic differences in the transduced cellular model (*i.e.*, human *vs.* rodent cell lines, *e.g.*, [13]), which may display a variable requirement of DNA damage signaling pathways during retroviral infection, as well as to the characteristics of the virus (*i.e.*, non-replicative form *vs.* replicative form which may contain encapsidated Ku, *e.g.*, [26]).

In addition, we confirmed the finding previously reported by Zheng and colleagues [27] on a positive role of encapsidated Ku on the retroviral cycle. Indeed, retroviral particles produced in a Ku-deficient context were transduced less efficiently in both WT and *Ku80^+/−^* target cells as compared to those produced in Ku-proficient cells. Noteworthy, the positive effect of exogenous Ku on retroviral cycle was not specific of the HIV-1, as it was also observed also for the SIN-PGK vector.

Also, we put in evidence a two-fold reduction of early HIV-1 transcription in human Ku80-haplodeficient HCT 116 cells as compared to their Ku-proficient counterparts. Of note, in all these experiments, the transducing virions were produced in Ku80-proficient cells (HEK 293T), as to ensure the specificity of the effect observed. Reportedly, Ku displays both a positive and a negative impact on the transcription of multiple genes, and this via direct interaction with their promoters *in vivo* and *in vitro* (*e.g.*, [36], [37], [38], [39]). Concerning HIV-1 expression, previous reports ascribed opposite effects to Ku. Indeed, Ku has been shown to have a positive impact on HIV-1 expression by acting in a Tat-dependent fashion [14]. In striking opposition to this observation, we previously reported a negative impact of Ku on HIV-1 expression in human U1 cells [12], a cellular model of pseudo-latency, as these cells contain two forms of HIV-1 proviruses mutated in the *tat* gene for avoiding their expression/replication in basal condition [49]. Hence, in the experimental setting of our previous study, cells already harboring mutated HIV-1 were subjected to Ku depletion, while the expression of latent HIV-1 proviruses required the obligate administration of TNFα [12]. Here, we demonstrated that Ku has a positive effect on HIV-1 short-term expression in HCT 116 cells, which are not a cellular model of pseudo-latency (*i.e.*, they do not bear any latent/mutated HIV-1 provirus). This effect was specific of the HIV-1 promoter and independent on HIV-1 regulatory and accessory proteins. It should be noted that in the present study, the depletion of Ku did alter the HIV-1 expression only when performed before virus transduction. Moreover, we showed that the positive effect of Ku on HIV-1 expression was independent from the binding to the putative KBS of HIV-1, as demonstrated by the finding that transgene expression from XCD24 Sc^-216-196^ (a vector carrying a scrambled sequence in place of the KBS), was still lower in Ku80-haplodepleted cells as compared to their WT counterparts. On the contrary, we provided some preliminary results on direct binding of Ku to a HIV-1 5'LTR region. Remarkably, Ku may bind to this site to a level similar to that observed for NF-κB on the HIV-1 LTR (*i.e.*, 3–12% depending of the cell line) [50], thus suggesting that Ku potentially acts as a transcription factor of the HIV-1 promoter. This hypothesis is in line with the notion that Ku is implicated in the transcriptional regulation of various genes including *IL2* [36] and MMTV [38], [39]. As proposed above, the apparent contradiction between the results presented in this manuscript and our previous observations could be ascribed to the different experimental setting used in the two studies, and namely in the cellular model (non pseudo-latent HCT 116 *vs.* pseudo-latent U1 cells), the characteristics of the transduced virus (non-replicative *vs.* latent), the time elapsed from transduction (early time *vs.* latency) the endpoint analyzed (HIV-1 expression *vs.* reactivation from latency) and the method of depletion of Ku (before *vs.* upon transduction).

Here, we provide evidence that the binding of Ku to HIV-1 LTR may stimulate HIV-1 transcription by associating to a specific region of its promoter. The obligatory requirement of virus integration shown in the present study seems to corroborate this hypothesis. Nevertheless, we could not rule out that Ku may also act via other alternative - but not mutually exclusive - mechanisms. Theoretically, Ku may promote early transcription from the LTR by either targeting the virus to specific host chromatin regions characterized by high transcriptional levels, or, alternatively, by contributing to the establishment of a local environment favorable for the early transcription from the LTR. Consistently with this latter hypothesis, Ku was shown to interact with multiple proteins positively implicated in HIV-1 transcription initiation, including DNA-PK, E26 transformation-specific 1 (ETS-1), NF-κB, RNA polymerase II, SP1 as well as in HIV-1 transcription elongation, such as the subunit cyclin-dependant kinase 9 (CDK9) of pTEFb [8], [51], [52], [53], [54], [55]. Moreover, Ku was reported to compete with direct HIV-1 inhibiting factors, such as nuclear factor 90 (NF90) [36] and RAD52 [56], or with indirect HIV-1 inhibiting factors, such as octamer-binding transcription factor 1 (OCT-1) [57].

Unfortunately, we were unable to re-express Ku in *Ku80^+/−^* HCT 116 cells and this is in contrast with previous findings [34]. We speculate that the level of Ku is regulated in *Ku80^+/−^* HCT 116 cells by a mechanism that either controls protein levels or negatively affects the survival/proliferation of cells expressing excessive amounts of Ku. In agreement with this hypothesis, the overexpression of Ku80 is reported to induce the activation of apoptosis in human hepatocellular carcinoma [58]. Intriguingly, p53 seems to contribute to this hypothetical process. Indeed, we put in evidence a cross-regulation between the expression levels of Ku and p53, a mutual relationship that was first anticipated by Hendrickson group [33], [34]. As both human *Ku80^+/−^* and murine *Ku80^−/−^* cells have been reported to have high level of spontaneous DNA lesions and/or DNA damage response [33], [34], [59], an appealing hypothesis would be that this hypothetical mechanism could act by limiting the potential deleterious effects caused by endogenous DNA damages [33], [34]. Remarkably, in our study, this Ku80/p53 interlinked control also modulates HIV-1 expression. These preliminary results, which call for further investigations, suggest that both the DNA repair machinery and p53 proficiency/level may influence HIV-1-driven expression. We believe that this notion has to be taken into account in choosing the cellular model to study HIV-1 replication cycle. As examples, p53 is mutated in CEM, Jurkat and U1 cell lines (database: http://www-p53.iarc.fr) and p53 functions are altered by the human papillomavirus E6 protein in HeLa cells [60] or by the human T-lymphotropic virus type 1 (HTLV-1) Tax oncoprotein in C8166 cells [27].

Finally, we showed that the depletion of Ku also promotes the establishment and/or the maintenance of viral latency in early times upon transduction, and this only when performed prior of HIV-1 transduction. Our results demonstrate that Ku limits viral latency by positively stimulating the expression of HIV-1. The mechanism underlying the establishment of latency is poorly known. Some recent evidence suggests that a limited availability of the host cell transcription initiation and elongation factors is associated to the establishment and/or the maintenance of viral latency [15], [16], [44]. These observations are in line with our hypothesis linking the absence of Ku leads with the occurrence of viral latency.

**Figure 7:**
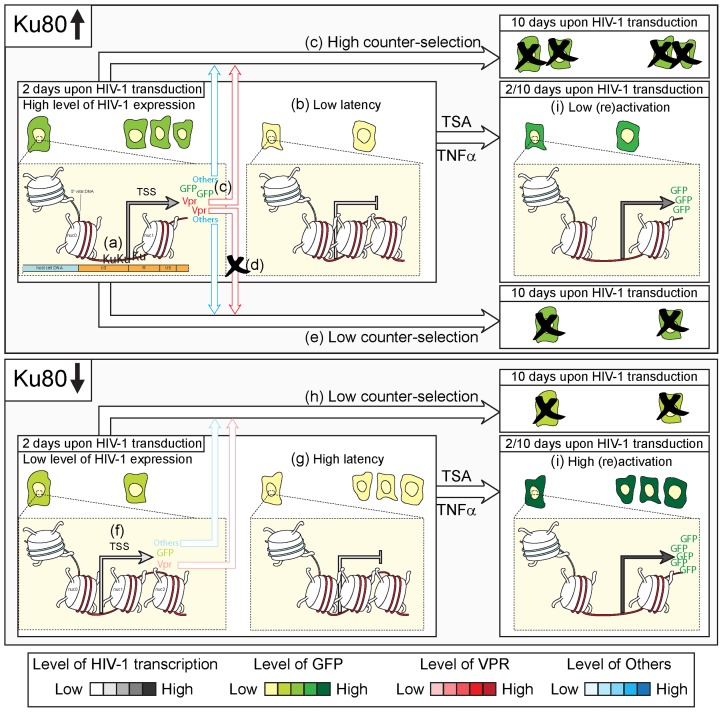
Hypothetical role of Ku on HIV-1 expression and latency at early- and mid-time upon transduction. When expressed at a normal level (as in Ku80-proficient cells), Ku stimulates the early expression of human immunodeficiency virus type 1 (HIV-1) by a direct binding to long terminal repeat (LTR) (**a**), a condition that is accompanied by a low level of viral latency (**b**) and that in turn leads to a high counter-selection of transduced cells over time for the expression of toxic viral proteins including Vpr (**c**). In line with this, the absence of Vpr (as by transducing the HIV-1 vector XCD3 *vpr^-^*) (**d**) decreases the level of cell death and/or counter-selection over time (**e**). On the contrary, a low level of Ku (as in *Ku80^+/−^* cells or in WT cells transfected with Ku-depleting siRNAs) negatively affects the expression of HIV-1 (**f**), increases the proportion of cells with latent viruses (**g**) and reduces the counter-selection phenomenon (**h**). Accordingly, the reactivation of HIV-1 upon trichostatin A (TSA)- or Tumor Necrosis Factor α (TNFα)-treatment at 2 or 10 days after transduction is more efficient in *Ku80^+/−^* cells that in their WT counterparts (**i**). Hence, we suggest that the depletion of Ku may favor the appearance/maintenance of cells harboring integrated proviruses in a latent and readily (re-)activatable state starting from the early phases from HIV-1 transduction.

In conclusion, we propose the following model to describe the action of Ku in response to HIV-1 transduction (**Figure 7**). When normally expressed in target cells, Ku may favor early HIV-1-driven transcription by a direct interaction with the provirus promoter (**Figure 7**). This higher level of transcription leads to (*i*) the limitation of the establishment and/or the maintenance of viral latency, and (*ii*) the expression of toxic viral proteins, including Vpr, which in turn induces cell death and/or the counter-selection of HIV-1 expressing cells, and consequently, a lower level of viral reactivation at day 10 by TSA and TNFα (**Figure 7**). Accordingly, Vpr may induce apoptosis in HIV-1 infected cells, as demonstrated in multiple studies [61], [62], [63]. Conversely, in the absence of Ku, the HIV-1-driven transcription is negatively affected at an early time upon transduction, when a higher proportion of cells harbors silenced/poorly expressed proviruses (**Figure 7**). However, these cells bearing an integrated provirus are less subjected to counter-selection over time and display an elevated capacity to be reactivated by TNFα or TSA administration (**Figure 7**).

Given its multiple roles in HIV-1 replication cycle, we surmise that Ku could be a novel promising target in the context of anti-retroviral therapy. Further experiments are required to confirm this hypothesis.

## Materials and Methods

### Plasmids and Vector Production

The genetic organization of the LV vectors is presented in **Figure 1**. RRLSIN.cPPT.hPGK-gfp (shortened to “SIN-PGK”) is a SIN-LV vector containing the *enhanced gfp* transgene under the control of the human PGK internal promoter. SIN-PGK was generated as previously described [64]. pXCD3 is derived from pNL4.3 containing the HIV-1 genome (GenBank: AF324493.1) from which the *Nhe*I-*Bsa*BI region of the *env* gene was deleted (named HIV-1 NL4.3 *env^-^*) and the *Hpa*I-*Xho*I fragment (corresponding to the *nef* gene) was replaced by the compatible IRES-*humanized Renilla gfp* (*hr-gfp*) fragment from the pIRES-hrGFP 1a plasmid (Clontech Laboratories, Inc., Mountain View, CA, USA). pXCD3 IN(D116A) is an integrase-defective version of pXCD3 encoding a mutated (D116A) integrase. This mutation was introduced into pXCD3 by directed PCR mutagenesis produced using the QuikChange XL Site-Directed Mutagenesis Kit (Agilent Technologies, Inc., Santa Clara, CA, USA) according to manufacturer's indications and with OBM156/OBM157 primers (**Table S2**). HR’-CMV-gfp (shortened to “HR’-CMV”) is an LV vector containing the *gfp* gene under the control of the immediate early human CMV and U3 LTR promoters [65]. pXCD24 was derived from pHR’-CMV by deleting the *Cla*I-*Bam*HI fragment corresponding to the CMV promoter. The pXCD24 TAR(C37T) construct was generated by replacing the original *Kpn*I-*Xba*I 3′LTR fragment of pXCD24 by a TAR(C37T) mutated version. This latter was obtained after insertion of the original *Kpn*I-*Xba*I 3′LTR fragment into the pGem-T-easy vector (Promega Corporation, Madison, WI, USA) and directed mutagenesis (as mentioned above) with the OBM200/OBM201 primers (**Table S2**). The pXCD24 Sc^-216-196^ construct was obtained by replacing the putative KBS (CGGAGAGAGAAGTATTAGAGT) located between nucleotides -216 and -196 of the *Kpn*I-*Xba*I U3 3′LTR region of pXCD24 by a scrambled sequence (ACTCTCCTAGGTCTACTTGAC). This latter was generated after insertion of the *Kpn*I-*Xba*I 3′LTR region of pXCD24 into the pGem-T-easy vector (Promega) followed by fusion PCR. In this aim, two separate PCRs were performed on pXCD24 by using the OBM101/OBM102 primers (**Table S2**) for the first PCR, and the OBM103/OBM104 primers (**Table S2**) for the second PCR, following the manufacturer’s recommendations for *Taq* Platinium High Fidelity amplification reactions (Invitrogen, Life Technologies Co., Carlsbad, CA, USA). Then, 1/50 of each PCR product was mixed together to perform a third PCR without primers for the first 10 cycles (98°C for 10s/45°C for 10s/72°C for 30s), and with the OBM101/OBM104 primers (**Table S2**) for the last 20 amplification cycles (98°C for 10 s/60°C for 10 s/72°C for 30 s). The resulting PCR product was inserted into the pGem-T-easy vector (Promega) according to the manufacturer’s recommendations, thereby generating the *Kpn*I-*Xba*I 3′LTR mutated fragment containing the scrambled sequence. The *Kpn*I-*Xba*I 3′LTR mutated fragment containing the scrambled sequence in place of the putative KBS was reintroduced into pXCD24 to replace the original *Kpn*I-*Xba*I region, thereby generating the pXCD24 Sc^-216-196^ construct. All constructs were maintained and stored in the Sure2 *E. coli* host strain (Agilent Technologies). LV vectors were generated by calcium phosphate-mediated transfection as detailed in [64].

### Cell Culture

WT and *Ku80^+/−^*70.32 human colorectal carcinoma epithelial HCT 116 cells (kindly provided by Dr E.A. Hendrickson) [33] were maintained in McCoy’s 5A medium. *TP53^−/−^* HCT 116 cells as well as *Ku80^+/−^* HCT 116 derivatives containing either a neomycin (Neo) or a hygromycin-Ku80 expression pcDNA3.1 plasmid (named A6 clone) (shortened as ‘pCtl’ and ‘pKu80’, respectively) were obtained from Horizon Discovery Ltd (Cambridge, UK) and maintained in McCoy’s 5A medium (under a 10 µg/ml hygromycin selection for pKu80 *Ku80^+/−^* HCT 116 cells). Human HEK 293T cells were maintained in Dulbecco’s modified Eagle medium. Human T CD4^+^ lymphoid lymphoma SUP-T1 cells [66] as well as human T CD4^+^ lymphoid leukemia Jurkat cells [67] and CEM-T4 cells (a naturally isolated subclone of the CEM line displaying high levels of surface CD4 expression; obtained from J.P. Jacobs through the AIDS Research and Reference Reagent Program, Division of AIDS, National Institute of Allergy and Infectious Diseases, National Institutes of Health) [68] were cultured in RPMI-1640. All media, purchased from Gibco (Life Technologies Co.), were supplemented with 10% fetal bovine serum (PAA Laboratories GmbH, Pasching, Austria) and 1% penicillin/streptomycin (100 units/mL). All cell lines used in this study were incubated at 37°C, under an atmosphere containing 5% CO_2_.

### Transduction and Transfection Procedures

The day before the transduction, 10^5^ cells/well were plated in 12-well plates. Transduction was performed by addition of the viral suspension to each cell line depending on its confluence estimated the day of transduction. Two days post-transduction, transgene expression was evaluated by cytofluorometry as described below. The m.o.i. was calculated according to the percentages of GFP^+^ cells observed during the linear phase of transduction. Alternatively, for reactivation experiments, cells were transduced and maintained in a 25 cm^2^ flask. Upon one week, 10^5^ cells/well were plated in 12-well plates and treated one day later with 200 nM of TSA (Sigma-Aldrich, St Louis, MO, USA) or 10 ng/ml of TNFα (BD Biosciences, San Diego, CA, USA) for additional 24 h. Dimethyl sulfoxide (DMSO, Sigma-Aldrich) was used as negative control of TSA treatment. For experiments exploring the reactivation of transgene expression in a pure population of transduced cells, GFP^+^ cells were sorted two or three days after transduction at low m.o.i. (<0.1). Cells were then cultured for six weeks and subjected every week to a 24 h TSA-treatment. For both reactivation experiments, transgene expression was finally determined by flow cytometry.

SiRNAs transfection was performed using custom-designed siRNAs duplexes (**Table S2**) targeting Ku80, Ku70, p53 encoding mRNA or a sequence unrelated to the human genome (Ctl) (all purchased from Eurogentec S.A., Seraing, Belgium) as previously described [69].

### Cytofluorometric Analysis

Transgene expression (*i.e.*, percentage and MFI of GFP^+^ cells) was estimated by flow cytometry using a FACSCalibur™ cytofluorometer (BD Biosciences). Analyses were carried out with CellQuestTM software (BD Biosciences), upon gating on the events characterized by normal forward/side scatter parameters. To assess cell cycle distribution, cells were stained and analyzed as previously described [69].

### Immunoblotting

Protein samples were prepared, separated, electrotransferred and immunoblotted using specific antibodies for Ku80, Ku70, p53 or β-actin [all from Santa Cruz Biotechnology, Inc., Santa Cruz, CA, USA; references (ref.) H-300, N3H10, DO-1, I-19, respectively], as previously described [69].

### Quantification of Viral DNA Species and of the Antigen p24

Two, 8, 24, 72 and 240 h post-transduction, cells were collected, centrifuged at 270 g and washed once with PBS. Thereafter, genomic DNA (gDNA) samples were prepared and Q-PCR was performed as previously described [64]. Primers and probes (**Table S2**) used for integrated vDNA nested PCR as well as LV total vDNA and 2-LTR amplification [performed on LightCycler (Roche Diagnostics, Basel, Switzerland)] are described in [35]. The cell equivalents in sample DNA as well as the copy number of total vDNA and integrated vDNA were calculated as previously described [64]. Alternatively, the copy number of 2-LTR circular vDNA forms was determined with reference to 10^2^ to 10^6^ copies of linearized pXCD3 plasmid or pGem-T-easy vector containing the XCD3 2-LTR sequence.

The quantification of HIV-1 p24 antigen in viral suspensions was performed using p24 HIV-1 antigen ELISA assays (PerkinElmer Life Sciences, Inc., Boston, MA, USA) following the manufacturer's recommendations.

### Chromatin Immunoprecipitation Assay

ChIP assays were performed with the chromatin immunoprecipitation assay kit (Upstate Biotechnology, EMD Millipore Corporation, Billerica, MA, USA), as previously described [70] and with the following modifications. Briefly, 10^7^ infected cells at 0.3–0.4 m.o.i (that correspond to 10^4^ copies of total vDNA/µg) were treated with 1% formaldehyde for 10 min at 37°C or 5 min at 4°C for the CEM-T4 or the SUP-T1 cell line, respectively. Cross-linked cells were harvested, washed with PBS, and lysed in sodium dodecyl sulfate (SDS) lysis buffer for 10 min at 4°C. Chromatin was sonicated (6 or 10 10-s pulses for the CEM-T4 or SUP-T1 cells, respectively) and centrifuged. Then, supernatants were diluted 10-fold with ChIP dilution buffer and precleared with salmon sperm DNA-protein A-agarose beads. Diluted extracts were kept (one tenth) for a direct Q-PCR (input DNA) or incubated with 1 µg/ml of specific antibodies for histone H3 or Ku80 (respectively from Upstate Biotechnology – ref. 06–755 or Santa Cruz Biotechnology – ref. H-300) followed by incubation with salmon sperm DNA-protein A-agarose beads (immunoprecipitated vDNA). Following extensive washing, bound vDNA fragments were eluted and the DNA was recovered by incubation in elution buffer and incubated with proteinase-K. The immunoprecipitated and input vDNA were then extracted and subjected to PCR quantification of LV total vDNA.

### Statistical Procedures

Unless otherwise specified, experiments were performed in triplicates and independently repeated at least twice. Data were analyzed with Microsoft Excel (Microsoft Co., Redmond, WA, USA) and statistical significance was assessed by means of two-tailed Student’s *t* test (*, *p*<0.05; **, *p*<0.01).

The full set of experimental procedures and statistical methods can be found online as Text S1.

## Supporting Information

Figure S1 **Characterization of WT and** ***Ku80^+/−^*** **HCT 116 cells.** (**A**,**B**,**D**) Wild-type (WT) and *Ku80^+/−^* human colon carcinoma HCT 116 cells were subjected to reverse transcription quantitative PCR (RT-Q-PCR) analysis (**A**), to evaluate the level of Ku80 encoding *xrcc5* mRNA and Western-blot assay (**B**,**D**), to assess Ku80, Ku70 and p53 protein contents. In panel (**A**), *xrcc5* mRNA content (normalized to that of *β-actin* mRNA) of WT HCT 116 cells was normalized to that of *Ku80^+/−^* cells (mean ± SD, n  = 3). (**C**) WT and *Ku80^+/−^* HCT 116 cells were stained with propidium iodide for the cytofluorometric assessment of cell cycle progression. Panel (**C**) reports cell cycle distributions and quantitative data of 2 independent experiments for both cell lines (mean ± SEM). In panels (**B**) and (**D**), actin levels were monitored to ensure equal loading of lanes.
(TIF)

Figure S2 **Analysis of HIV-1 expression in** ***TP53^−/−^*** **HCT 116 cells.** (**A**) Western-blot-mediated assessment of Ku80 protein contents in wild-type (WT) and *TP53^−/−^* human colon carcinoma HCT 116 cells. Actin levels were monitored to ensure equal loading of lanes. (**B**) HCT 116 cells with the illustrated genetic background were transduced with XCD3 (HIV-1 *env^-^ nef ^-^* IRES-*gfp*) at low m.o.i. (<0.3) for 48 h, followed by cytofluorimetric assessment of green fluorescent protein (GFP) expression (mean ± SEM, n  = 2; *, p<0.05).
(TIF)

Figure S3 **Characterization of a Ku** ***trans*** **-complemented** ***Ku80^+/−^*** **HCT 116 clone.** Wild-type (WT) and *Ku80^+/−^* human colon carcinoma HCT 116 cells as well as derivatives containing either a neomycin (Neo) or a hygromycin-Ku80 expression pcDNA3.1 plasmid (shortened as ‘pCtl’ and ‘pKu80’, respectively) were subjected to Western-blot assessment of Ku80 protein contents. Actin level was monitored to ensure equal loading of lanes. Please note that the pKu80 *Ku80^+/−^* HCT 116 clone was maintained or not under a 10 µg/ml hygromycin selection (noted as ‘H’).
(TIF)

Figure S4 **Cytofluorometric-mediated analysis of transgene expression in HCT 116, Jurkat and CEM-T4 cell lines.** Wild-type (WT) and *Ku80^+/−^* human colon carcinoma HCT 116 cells as well as the human T lymphoid leukemia Jurkat and CEM-T4 cell lines were transduced with the same volume of XCD3 (HIV-1 *env^-^ nef ^-^* IRES-*gfp*) at a m.o.i <0.2. Upon the depicted time from transduction, cells were subjected either to cytofluorimetric analysis, to measure green fluorescent protein (GFP) expression. Results obtained in 2 independent experiments are shown (mean ± SD).
(TIF)

Figure S5 **Impact of the depletion of Ku in virus producing cells on viral transduction.** (**A**) Schematic illustration of the experimental strategy adopted in this figure. (**B-F**) Human embryonic kidney (HEK) 293T transfected with an unrelated sequence (siCtl) or with siRNAs directed against Ku80 (siKu80) or Ku70 (siKu70), were subjected to Western-blot analysis at the depicted time (**B**, actin levels were monitored to ensure equal loading of lanes), or, alternatively, were used for vector productions (as described in Material and Methods) 24 h after transfection (**C**–**F**). Upon their production, XCD3 (HIV-1 *env^-^ nef ^-^* IRES-*gfp*) (**C**–**F**) or self-inactivating -phosphoglycerate kinase (SIN-PGK) (**C**,**D**,**F**) vectors were transduced in WT (**C**–**F**) or *Ku80^+/−^* (**E**,**F**) HCT 116 cells, followed by quantitative PCR (Q-PCR) analysis of total viral DNA (vDNA) (**C**) or cytofluorimetry-mediated assessment of green fluorescent protein (GFP) expression (**D**–**F**) 24 h or 48 h later, respectively. The quantity of total vDNA and the percentages of GFP-positive (GFP^+^) cells were normalized to the amount of the viral protein p24 per cell as determined by p24 quantification of vectors. In panels (**C**,**D**), the results are expressed as mean ± SEM (n  = 3). *, p<0.05 as compared to the HP/siCtl ratio in (**C**). *, p<0.05 as compared to WT cells subjected to HP and ^#^, p<0.05 as compared to siCtl-transfected WT cells in (**D**). In panel (**E**), one representative experiment (out of two independent ones yielding similar results) is shown (mean ± SD; *, p<0.05 as compared to WT cells subjected to HP; ^#^, p<0.05 as compared to siCtl-transfected WT cells). Panel (**F**) reports the depicted ratios of GFP^+^ cells between HP, siCtl or siKu70 conditions for WT and *Ku80^+/−^* HCT 116 cells transduced for 2 days with XCD3 or SIN-PGK [as obtained in (**D**,**E**)]. Two (XCD3) or one (SIN-PGK) representative titrations (out of at least two independent ones yielding similar results) of one viral production are illustrated (mean ± SD; *, p<0.05 as compared to the HP/siCtl ratio). HP (Hiperfect®) corresponds to cells kept in control condition or exposed to the same amount of liposomes used for transfection but in absence of siRNA.
(TIF)

Figure S6 **The transcription profiles of HERVs are similar in both WT and** ***Ku80^+/−^*** **HCT 116 cells.** Three independent pairs of total RNA from wild-type (WT) and *Ku80^+/−^* human colon carcinoma HCT 116 were compared by microarray hybridization. Images were saved in the Bitmap file format and were used to present the results by false color mapping. Each hybridization result is representative of a triplicate yielding similar results. Grey boxes indicate the four human endogenous retrovirus (HERV) taxons (HML-1, HML-3, HML-5 and HML-9) among 57 (24 groups of HERVs related to β-retrovirus, γ-retroviruses and spumaviruses) probed by RetroArrays that were identified by Biometric Research Branch (BRB)-ArrayTools-mediated analyses. These four HERV groups exhibited a small but significant variation of expression in WT *vs.* Ku80-haplodeficient cells (non parametric *p* value <0.01, intensity threshold fixed at ±30% of variation between the two cell lines, **Table S1**; for more details, see also text S1). However, subsequent reverse transcription quantitative PCR (RT-Q-PCR) experiments performed for the HERV groups exhibiting the most robust difference (namely, HML-1 and HML-5) did not confirm this result, which is probably due to a limited sensibility of the RetroArrays and the fact that the primers used for RT-Q-PCR do not match exactly the same subset of HERV sequences as the capture probes spotted on the RetroArrays (**Table S2**).
(PDF)

Figure S7 **The decrease of HIV-1-driven expression over time is reduced in the absence of Vpr.** (**A–C**) Wild-type (WT) and *Ku80^+/−^* human colon carcinoma HCT 116 cells were transduced with XCD3 expressing *vpr* (XCD3, HIV-1 *env^-^ nef ^-^* IRES-*gfp*), or its derivative lacking *vpr* (XCD3 *vpr^-^*, refer to **Figure 1** for more details on these vectors). Upon 2 and/or 10 days from transduction, the percentage of green fluorescent protein-positive (GFP^+^) cells was determined by flow cytometry (D2 and D10, respectively). (**A**) One representative experiment performed in triplicates (out of four independent ones yielding similar results) is shown (mean ± SD; *, p<0.05, as compared to transduced cells analyzed at D2). In panel (**B**), the ratios of GFP^+^ cells between WT and *Ku80^+/−^* HCT 116 cells 2 days after transduction with XCD3 or XCD3 *vpr^-^* [as obtained in (**A**)] are illustrated (mean ± SD, n  = 4). (**C**) Alternatively, upon 1 and/or 10 days from transduction, the quantity of total vDNA was determined by quantitative PCR (Q-PCR) analysis (D1 and D10, respectively) (mean ± SEM, n  = 2; *, p<0.05, as compared to XCD3-transduced cells).
(TIF)

Figure S8 **The depletion of Ku in WT HCT 116 cells after HIV-1 transduction has no impact on viral expression.** (**A**,**B**) Wild-type (WT) human colon carcinoma HCT 116 cells were transduced for 2 days with XCD3 (HIV-1 *env^-^ nef ^-^* IRES-*gfp*) at low m.o.i. (<0.1; *i.e.*, one provirus per cell), followed by 8 h of transfection with an unrelated sequence (siCtl) or with siRNAs directed against Ku80 (siKu80). Twenty-four and 48 h later, the protein level of Ku80 was assessed by Western-blot analysis (**A**). Actin levels were monitored to ensure equal loading of lanes. Alternatively, 72 h upon siRNA transfection, cells were subjected by cytofluorometry-mediated assessment of green fluorescent protein (GFP) expression 24 h after administration of trichostatin A (TSA) or not (**B**). Results from 2 independent productions are expressed as mean ± SEM. HP (Hiperfect®) corresponds to cells kept in control condition or exposed to the same amount of liposomes used for transfection but in absence of siRNA. Please refer to **Figure 3A** for the Western-blot analysis of the Ku80 protein level 72 h post-transfection.
(TIF)

Figure S9 **Ku80 haplodepletion does not increase the HIV-1 reactivation level in GFP^+^ transduced cells.** (**A**,**B**) Wild-type (WT) and *Ku80^+/−^* human colon carcinoma HCT 116 cells were transduced for 2 days with XCD3 (HIV-1 *env^-^ nef ^-^* IRES-*gfp*) at low m.o.i. (<0.1; *i.e.*, one provirus per cell), followed by the isolation of green fluorescent protein-positive (GFP^+^) cells by means of cytofluorometry-mediated cell sorting. Thereafter, GFP^+^ isolated cells were maintained in culture for 4 weeks and the percentages of GFP-expressing were routinely monitored by flow cytometry-mediated analysis, as indicated (**A**). Alternatively, 2 and 4 weeks after cell sorting, transduced cells were left untreated or exposed for 24 h to tricostatin A (TSA) (**B**). Columns in (**B**) represent percentages of GFP^+^ cells in each condition. Panel (**A**) shows results coming from at least 2 independent experiments (mean ± SEM), while panel (**B**) illustrates one representative experiment (out of 2 independent ones yielding similar results, mean ± SD).
(TIF)

Table S1 **HERV groups analyzed by RT-Q-PCR.**
(DOC)

Table S2 **Sequence of primers, probes and siRNA used for this study.**
(DOC)

Text S1 **Supplementary materials and methods.**
(DOC)
