# Supplementary figures and images for: Impact of the Ku Complex on HIV-1 Expression and Latency
Source: PLoS One. 2013 Jul 29;8(7):e69691. doi: 10.1371/journal.pone.0069691 (PMC3726783; doi:10.1371/journal.pone.0069691)

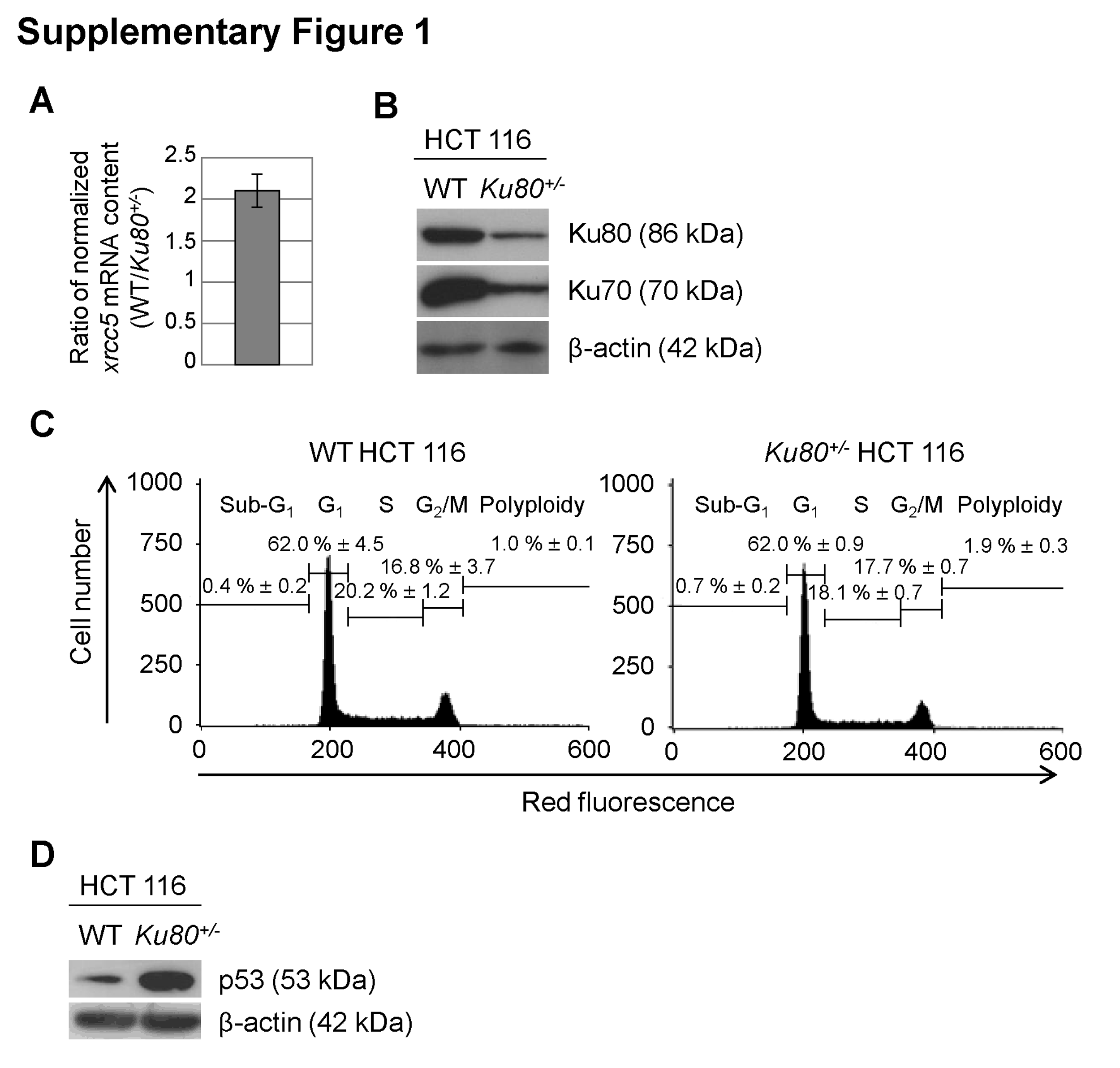

Supplement: Figure S1 — Characterization of WT and Ku80+/− HCT 116 cells. (A,B,D) Wild-type (WT) and Ku80+/− human colon carcinoma HCT 116 cells were subjected to reverse transcription quantitative PCR (RT-Q-PCR) analysis (A), to evaluate the level of Ku80 encoding xrcc5 mRNA and Western-blot assay (B,D), to assess Ku80, Ku70 and p53 protein contents. In panel (A), xrcc5 mRNA content (normalized to that of β-actin mRNA) of WT HCT 116 cells was normalized to that of Ku80+/− cells (mean ± SD, n = 3). (C) WT and Ku80+/− HCT 116 cells were stained with propidium iodide for the cytofluorometric assessment of cell cycle progression. Panel (C) reports cell cycle distributions and quantitative data of 2 independent experiments for both cell lines (mean ± SEM). In panels (B) and (D), actin levels were monitored to ensure equal loading of lanes. (TIF) [file pone.0069691.s001.tif]

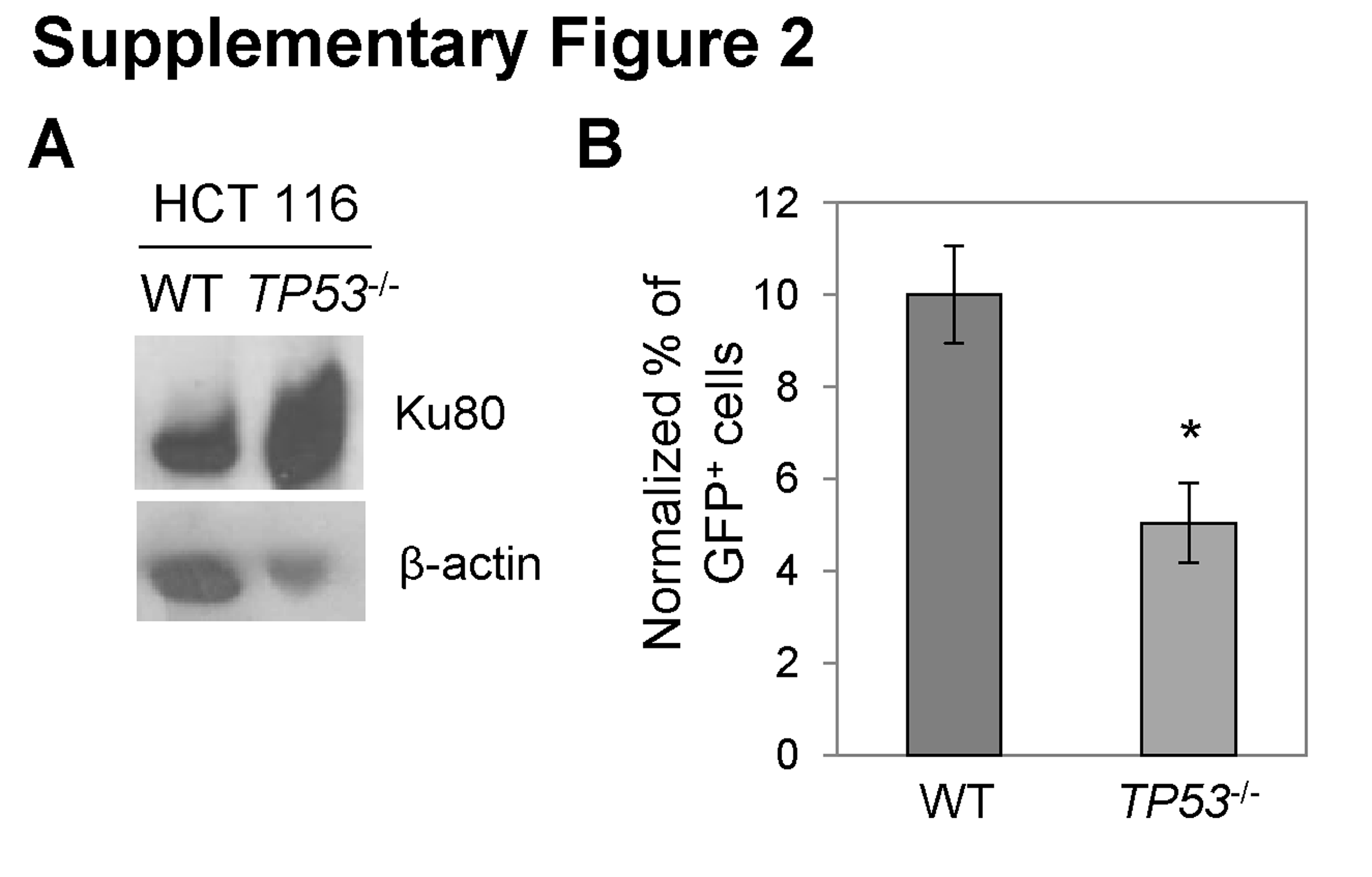

Supplement: Figure S2 — Analysis of HIV-1 expression in TP53−/− HCT 116 cells. (A) Western-blot-mediated assessment of Ku80 protein contents in wild-type (WT) and TP53−/− human colon carcinoma HCT 116 cells. Actin levels were monitored to ensure equal loading of lanes. (B) HCT 116 cells with the illustrated genetic background were transduced with XCD3 (HIV-1 env- nef - IRES-gfp) at low m.o.i. (<0.3) for 48 h, followed by cytofluorimetric assessment of green fluorescent protein (GFP) expression (mean ± SEM, n = 2; *, p<0.05). (TIF) [file pone.0069691.s002.tif]

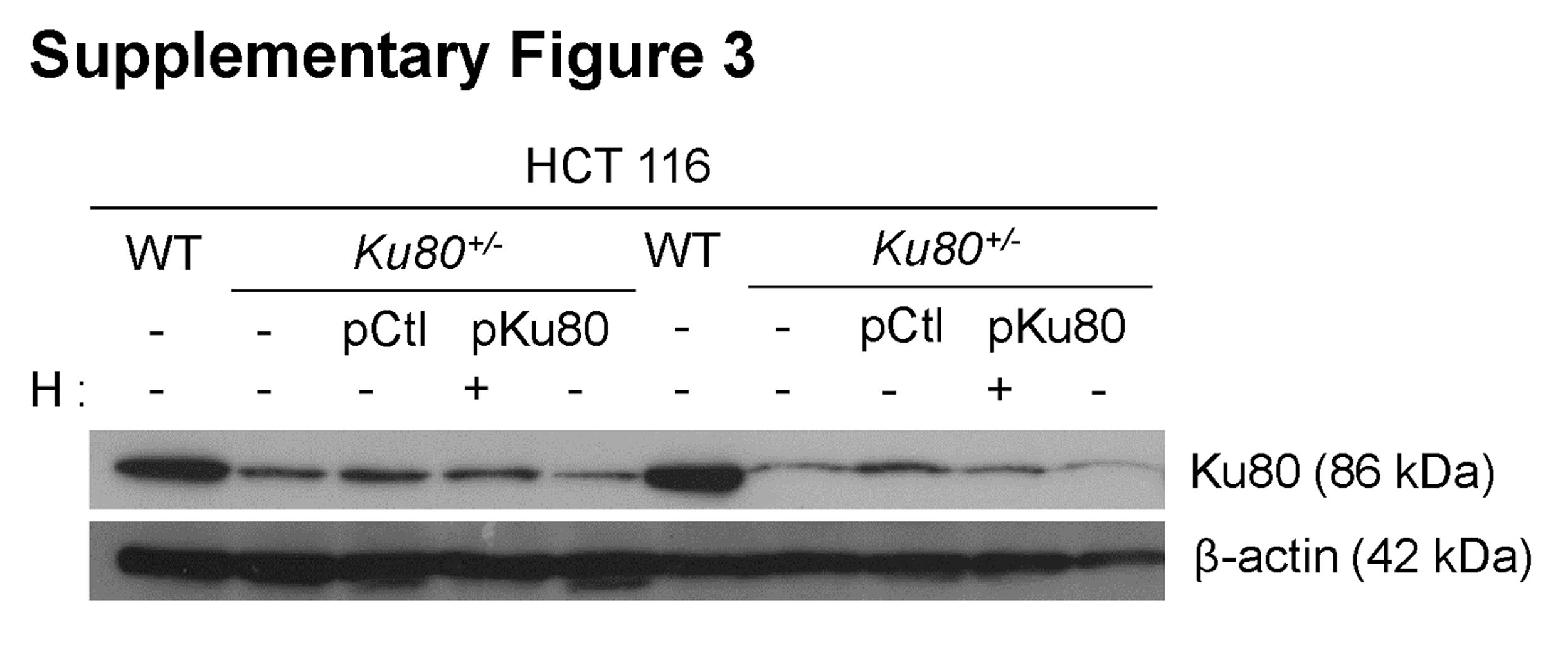

Supplement: Figure S3 — Characterization of a Ku trans -complemented Ku80+/− HCT 116 clone. Wild-type (WT) and Ku80+/− human colon carcinoma HCT 116 cells as well as derivatives containing either a neomycin (Neo) or a hygromycin-Ku80 expression pcDNA3.1 plasmid (shortened as ‘pCtl’ and ‘pKu80’, respectively) were subjected to Western-blot assessment of Ku80 protein contents. Actin level was monitored to ensure equal loading of lanes. Please note that the pKu80 Ku80+/− HCT 116 clone was maintained or not under a 10 µg/ml hygromycin selection (noted as ‘H’). (TIF) [file pone.0069691.s003.tif]

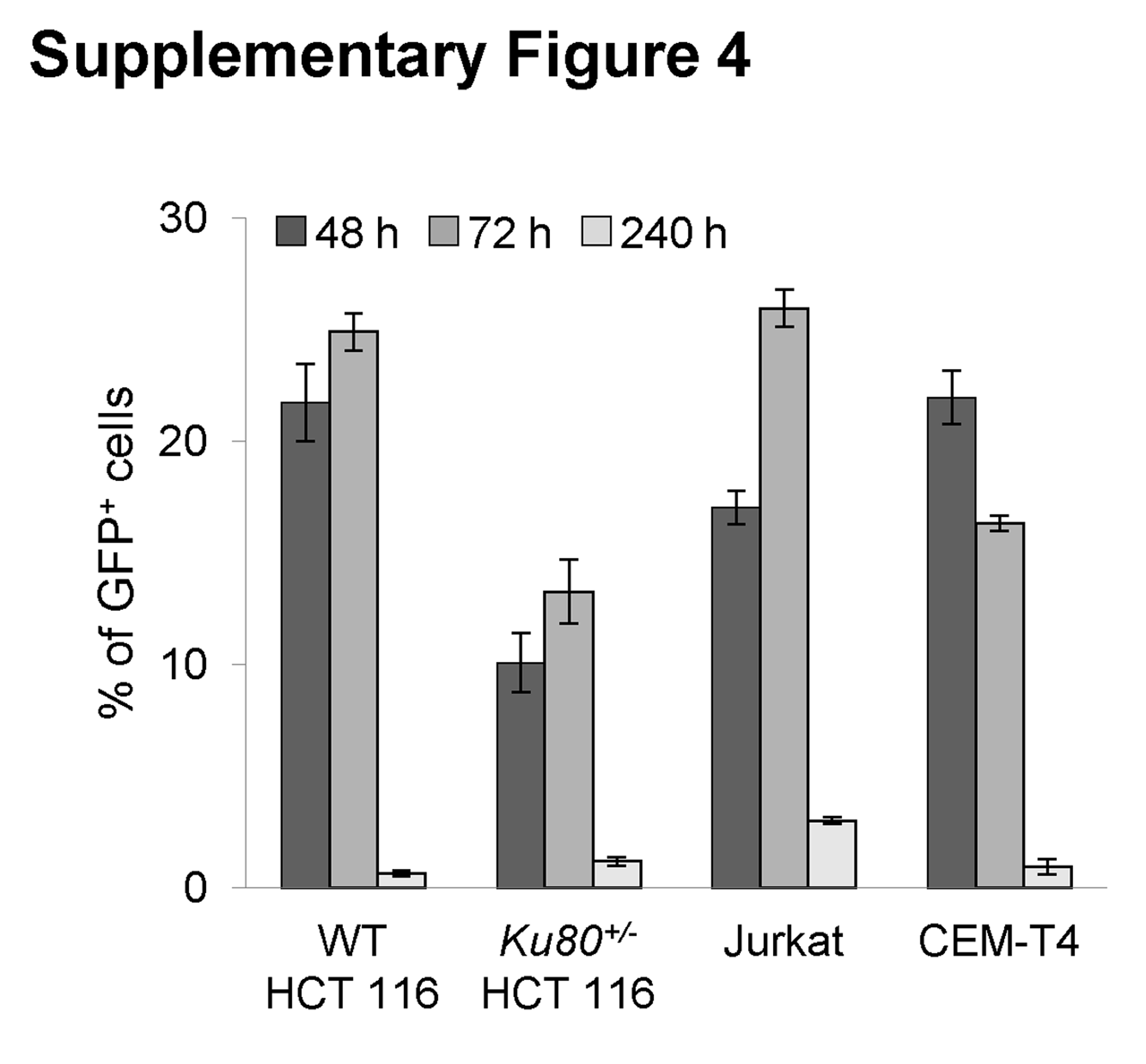

Supplement: Figure S4 — Cytofluorometric-mediated analysis of transgene expression in HCT 116, Jurkat and CEM-T4 cell lines. Wild-type (WT) and Ku80+/− human colon carcinoma HCT 116 cells as well as the human T lymphoid leukemia Jurkat and CEM-T4 cell lines were transduced with the same volume of XCD3 (HIV-1 env- nef - IRES-gfp) at a m.o.i <0.2. Upon the depicted time from transduction, cells were subjected either to cytofluorimetric analysis, to measure green fluorescent protein (GFP) expression. Results obtained in 2 independent experiments are shown (mean ± SD). (TIF) [file pone.0069691.s004.tif]

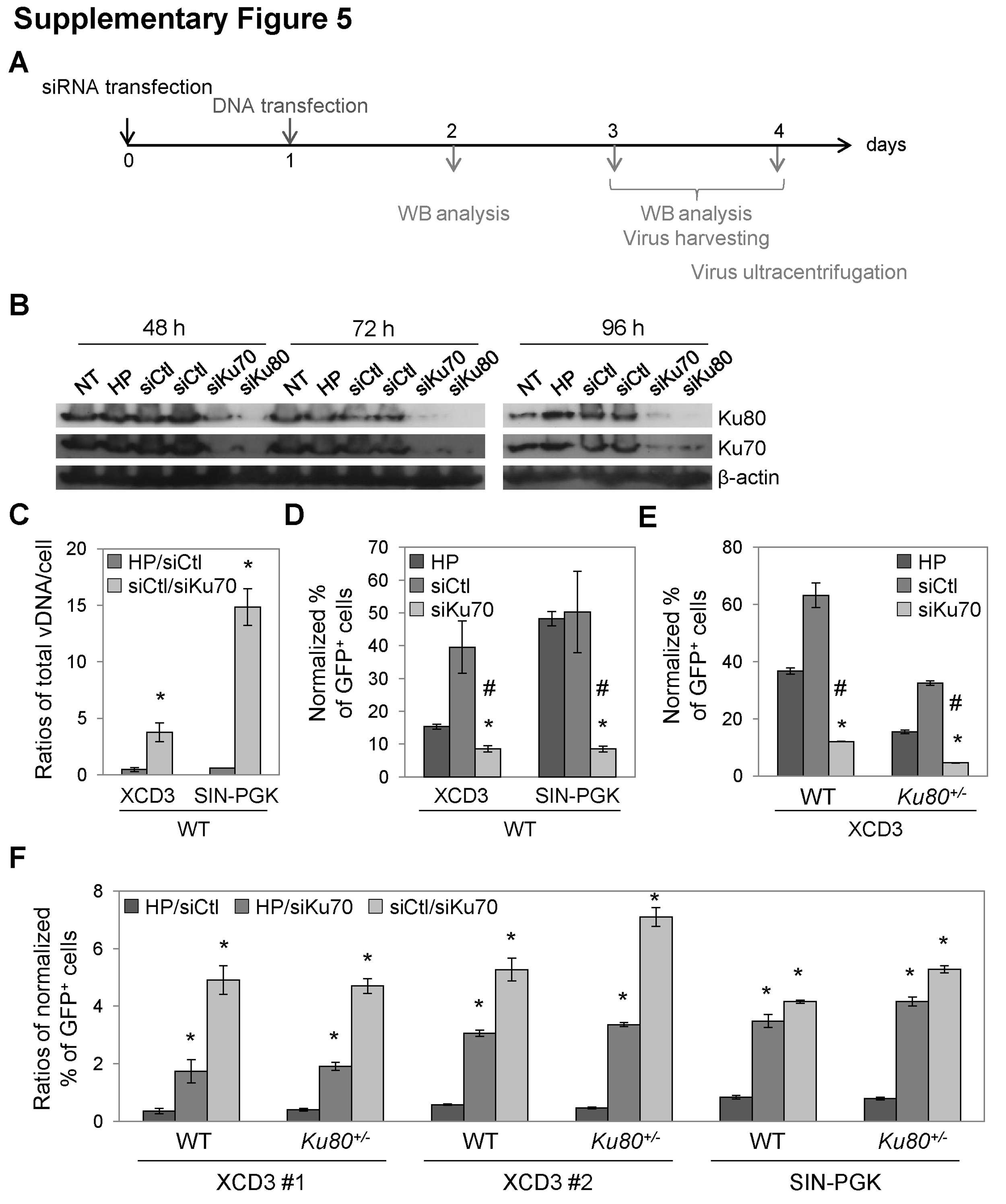

Supplement: Figure S5 — Impact of the depletion of Ku in virus producing cells on viral transduction. (A) Schematic illustration of the experimental strategy adopted in this figure. (B-F) Human embryonic kidney (HEK) 293T transfected with an unrelated sequence (siCtl) or with siRNAs directed against Ku80 (siKu80) or Ku70 (siKu70), were subjected to Western-blot analysis at the depicted time (B, actin levels were monitored to ensure equal loading of lanes), or, alternatively, were used for vector productions (as described in Material and Methods) 24 h after transfection (C–F). Upon their production, XCD3 (HIV-1 env- nef - IRES-gfp) (C–F) or self-inactivating -phosphoglycerate kinase (SIN-PGK) (C,D,F) vectors were transduced in WT (C–F) or Ku80+/− (E,F) HCT 116 cells, followed by quantitative PCR (Q-PCR) analysis of total viral DNA (vDNA) (C) or cytofluorimetry-mediated assessment of green fluorescent protein (GFP) expression (D–F) 24 h or 48 h later, respectively. The quantity of total vDNA and the percentages of GFP-positive (GFP+) cells were normalized to the amount of the viral protein p24 per cell as determined by p24 quantification of vectors. In panels (C,D), the results are expressed as mean ± SEM (n = 3). *, p<0.05 as compared to the HP/siCtl ratio in (C). *, p<0.05 as compared to WT cells subjected to HP and #, p<0.05 as compared to siCtl-transfected WT cells in (D). In panel (E), one representative experiment (out of two independent ones yielding similar results) is shown (mean ± SD; *, p<0.05 as compared to WT cells subjected to HP; #, p<0.05 as compared to siCtl-transfected WT cells). Panel (F) reports the depicted ratios of GFP+ cells between HP, siCtl or siKu70 conditions for WT and Ku80+/− HCT 116 cells transduced for 2 days with XCD3 or SIN-PGK [as obtained in (D,E)]. Two (XCD3) or one (SIN-PGK) representative titrations (out of at least two independent ones yielding similar results) of one viral production are illustrated (mean ± SD; *, p<0.05 as compared to the HP/siCtl ra [file pone.0069691.s005.tif]

Supplementary Figure 6

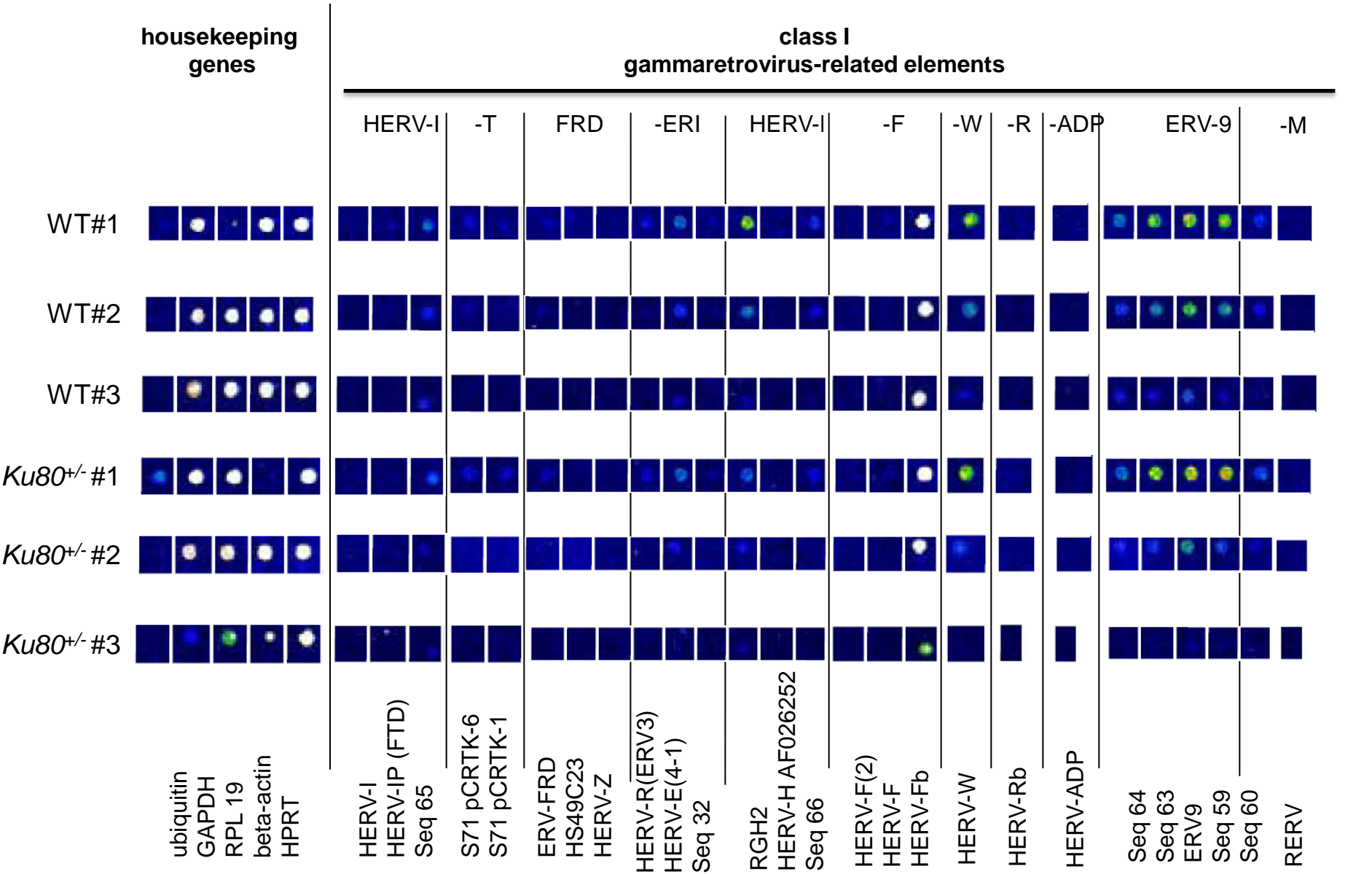

Supplementary Figure 6 (continued)

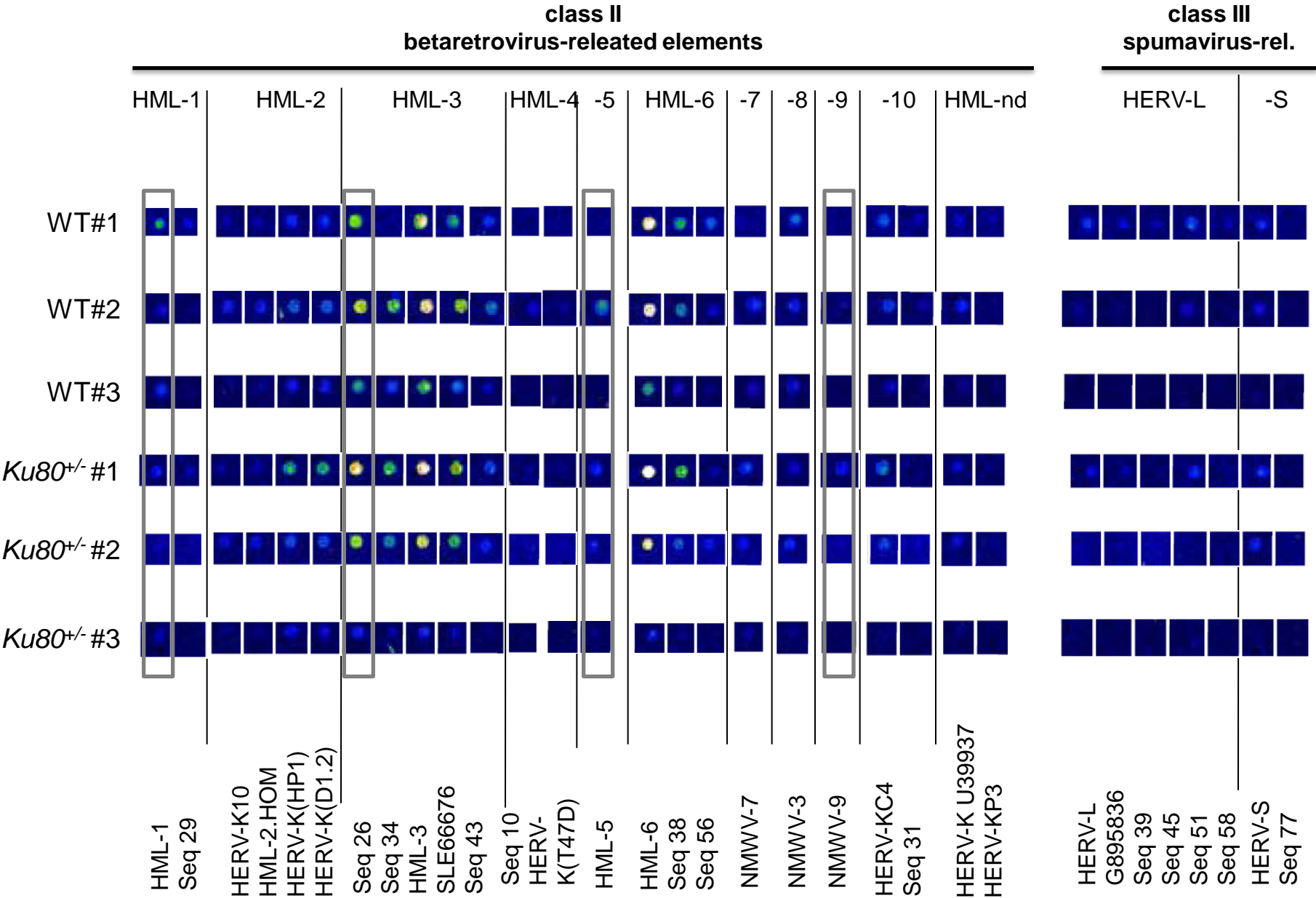

Supplement: Figure S6 — The transcription profiles of HERVs are similar in both WT and Ku80+/− HCT 116 cells. Three independent pairs of total RNA from wild-type (WT) and Ku80+/− human colon carcinoma HCT 116 were compared by microarray hybridization. Images were saved in the Bitmap file format and were used to present the results by false color mapping. Each hybridization result is representative of a triplicate yielding similar results. Grey boxes indicate the four human endogenous retrovirus (HERV) taxons (HML-1, HML-3, HML-5 and HML-9) among 57 (24 groups of HERVs related to β-retrovirus, γ-retroviruses and spumaviruses) probed by RetroArrays that were identified by Biometric Research Branch (BRB)-ArrayTools-mediated analyses. These four HERV groups exhibited a small but significant variation of expression in WT vs. Ku80-haplodeficient cells (non parametric p value <0.01, intensity threshold fixed at ±30% of variation between the two cell lines, Table S1; for more details, see also text S1). However, subsequent reverse transcription quantitative PCR (RT-Q-PCR) experiments performed for the HERV groups exhibiting the most robust difference (namely, HML-1 and HML-5) did not confirm this result, which is probably due to a limited sensibility of the RetroArrays and the fact that the primers used for RT-Q-PCR do not match exactly the same subset of HERV sequences as the capture probes spotted on the RetroArrays (Table S2). (PDF) [file pone.0069691.s006.pdf]

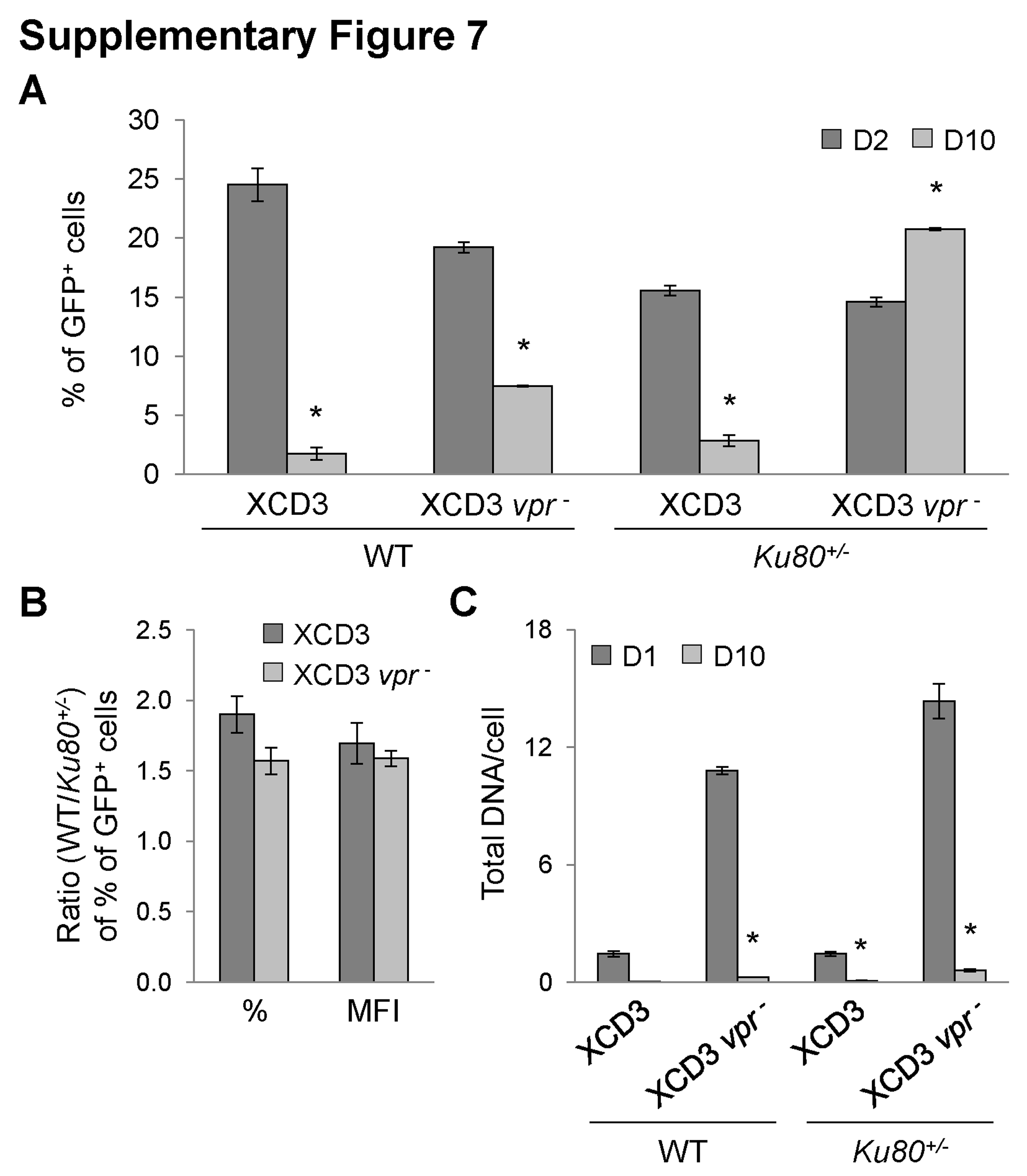

Supplement: Figure S7 — The decrease of HIV-1-driven expression over time is reduced in the absence of Vpr. (A–C) Wild-type (WT) and Ku80+/− human colon carcinoma HCT 116 cells were transduced with XCD3 expressing vpr (XCD3, HIV-1 env- nef - IRES-gfp), or its derivative lacking vpr (XCD3 vpr-, refer to Figure 1 for more details on these vectors). Upon 2 and/or 10 days from transduction, the percentage of green fluorescent protein-positive (GFP+) cells was determined by flow cytometry (D2 and D10, respectively). (A) One representative experiment performed in triplicates (out of four independent ones yielding similar results) is shown (mean ± SD; *, p<0.05, as compared to transduced cells analyzed at D2). In panel (B), the ratios of GFP+ cells between WT and Ku80+/− HCT 116 cells 2 days after transduction with XCD3 or XCD3 vpr- [as obtained in (A)] are illustrated (mean ± SD, n = 4). (C) Alternatively, upon 1 and/or 10 days from transduction, the quantity of total vDNA was determined by quantitative PCR (Q-PCR) analysis (D1 and D10, respectively) (mean ± SEM, n = 2; *, p<0.05, as compared to XCD3-transduced cells). (TIF) [file pone.0069691.s007.tif]

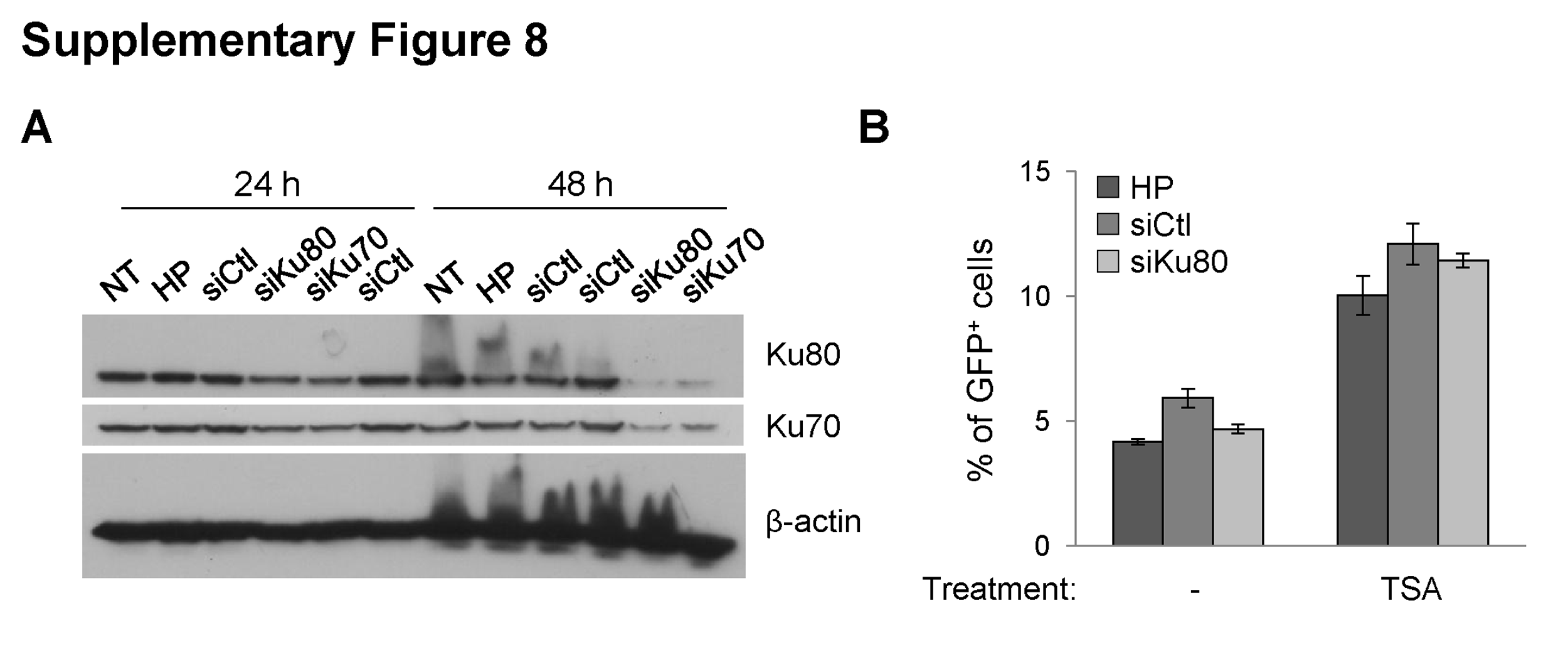

Supplement: Figure S8 — The depletion of Ku in WT HCT 116 cells after HIV-1 transduction has no impact on viral expression. (A,B) Wild-type (WT) human colon carcinoma HCT 116 cells were transduced for 2 days with XCD3 (HIV-1 env- nef - IRES-gfp) at low m.o.i. (<0.1; i.e., one provirus per cell), followed by 8 h of transfection with an unrelated sequence (siCtl) or with siRNAs directed against Ku80 (siKu80). Twenty-four and 48 h later, the protein level of Ku80 was assessed by Western-blot analysis (A). Actin levels were monitored to ensure equal loading of lanes. Alternatively, 72 h upon siRNA transfection, cells were subjected by cytofluorometry-mediated assessment of green fluorescent protein (GFP) expression 24 h after administration of trichostatin A (TSA) or not (B). Results from 2 independent productions are expressed as mean ± SEM. HP (Hiperfect®) corresponds to cells kept in control condition or exposed to the same amount of liposomes used for transfection but in absence of siRNA. Please refer to Figure 3A for the Western-blot analysis of the Ku80 protein level 72 h post-transfection. (TIF) [file pone.0069691.s008.tif]

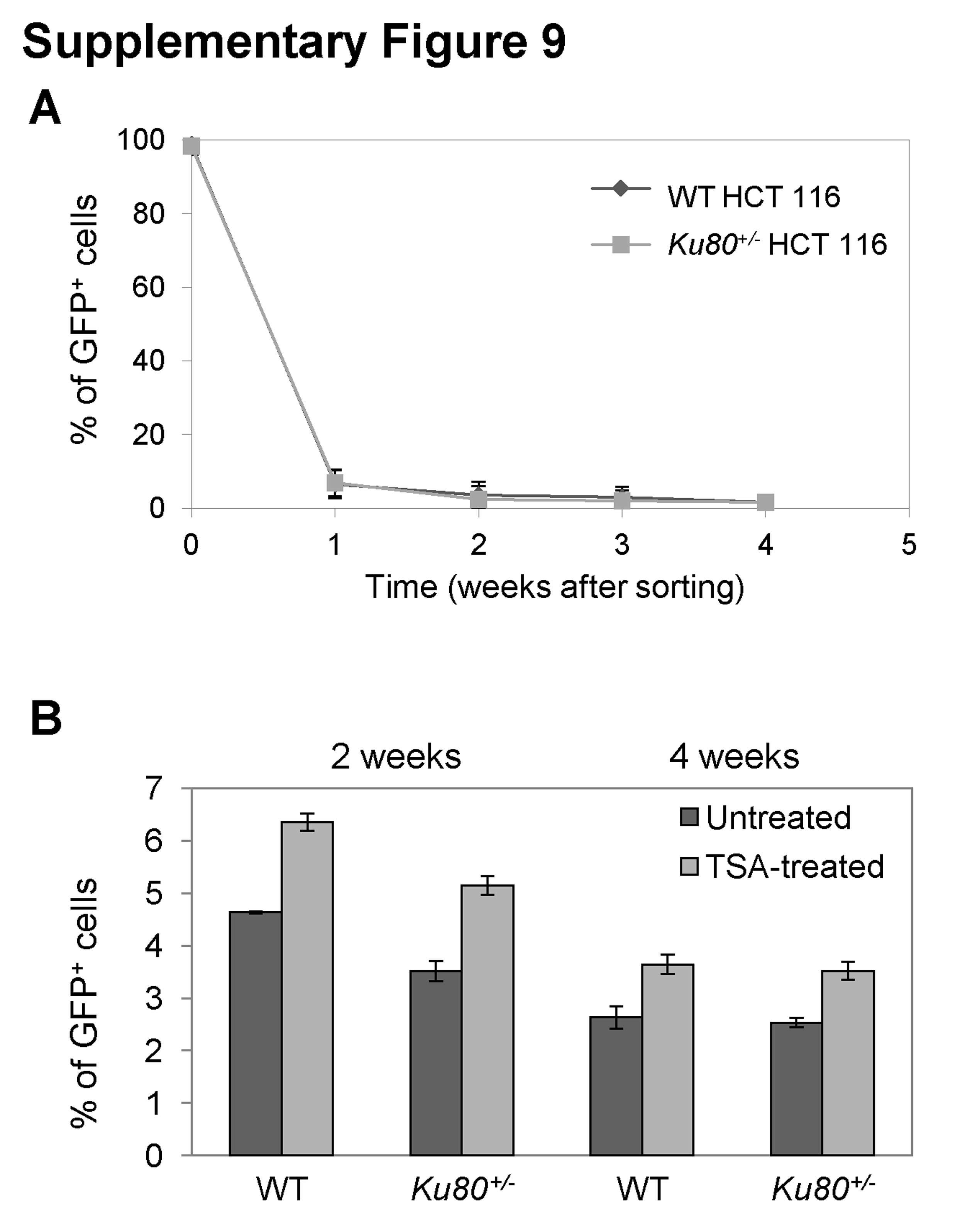

Supplement: Figure S9 — Ku80 haplodepletion does not increase the HIV-1 reactivation level in GFP+ transduced cells. (A,B) Wild-type (WT) and Ku80+/− human colon carcinoma HCT 116 cells were transduced for 2 days with XCD3 (HIV-1 env- nef - IRES-gfp) at low m.o.i. (<0.1; i.e., one provirus per cell), followed by the isolation of green fluorescent protein-positive (GFP+) cells by means of cytofluorometry-mediated cell sorting. Thereafter, GFP+ isolated cells were maintained in culture for 4 weeks and the percentages of GFP-expressing were routinely monitored by flow cytometry-mediated analysis, as indicated (A). Alternatively, 2 and 4 weeks after cell sorting, transduced cells were left untreated or exposed for 24 h to tricostatin A (TSA) (B). Columns in (B) represent percentages of GFP+ cells in each condition. Panel (A) shows results coming from at least 2 independent experiments (mean ± SEM), while panel (B) illustrates one representative experiment (out of 2 independent ones yielding similar results, mean ± SD). (TIF) [file pone.0069691.s009.tif]
