# Supplementary material for: Impact of the Ku Complex on HIV-1 Expression and Latency
Source: PLoS One. 2013 Jul 29;8(7):e69691. doi: 10.1371/journal.pone.0069691 (PMC3726783; doi:10.1371/journal.pone.0069691)
Supplement: Table S1 — HERV groups analyzed by RT-Q-PCR. (DOC) [file pone.0069691.s010.doc]

**Table S1: HERV groups analyzed by RT-Q-PCR**

| **Condition: WT HCT 116 versus *Ku80^+/-^* HCT 116** | | | | | | |
| --- | --- | --- | --- | --- | --- | --- |
| **Locus** | **Class** | **Parametric analysis on Norm2 data**  (Ratio, Student’s *t* test) | | **BrB analysis** | | **RT-Q-PCR results:**  Ratio WT/*Ku80^+/-^*  (± SD)  2 independent experiments realized on each RNA extract |
|  |  | **Ratio WT/*Ku80^+/-^***(Norm1 data) | ***P* value** | **Norm1 Data**  (parametric *p* value or «- »  if p>0,01) | **Norm3 data**  (parametric *p* value or «- »  if p>0,01) |  |
| ERV-FRD | γ | 0,7 | 0,00002 | - | - | 0,8 (± 0,2) |
| ERV9 (seq59) | γ | 0,7 | 0,009 | - | - | nd |
| HML-2 HERV-K (HP1) | β | 0,7 | 0,003 | - | - | 0,8 (± 0,3) |
| HERV-S | spuma | 0,6 | 0,005 | - | - | nd |
| ERI HERV-R (ERV3) | γ | 0,7 | 0,001 | - | - | nd |
| HML-7 MMWV-7 | β | 0,6 | 0,0008 | - | - | nd |
| **HML-9 MMWV-9** | **β** | **0,6** | **0,000001** | **0,001** | **0,006** | ns |
| HML-4 (Seq10) | β | 0,7 | 0,0000003 | - | - | nd |
| **HML-5** | **β** | **0,4** | **0,00053** | **0,009** | **0,003** | 0,7 (± 0,3) |
| **HML-1** | **β** | **1,6** | **0,008** | **0,007** | **-** | 0,8 (± 0,3) |
| **HML-3 (Seq26)** | **β** | **0,8** | **0,006** | **-** | **0,09** | nd |
| Actin |  |  |  |  |  | 1,0 (± 0,2) |

nd (not determined)

ns (non specific signal of RT-Q-PCR)

Abbreviations: BrB, Biometric Research Branch; HERV, human endogenous retrovirus; RT-Q-PCR, reverse transcription quantitative PCR; WT, wild-type
