# Supplementary material for: Impact of the Ku Complex on HIV-1 Expression and Latency
Source: PLoS One. 2013 Jul 29;8(7):e69691. doi: 10.1371/journal.pone.0069691 (PMC3726783; doi:10.1371/journal.pone.0069691)
Supplement: Table S2 — Sequence of primers, probes and siRNA used for this study. (DOC) [file pone.0069691.s011.doc]

**Table S2: Sequence of primers, probes and siRNA used for this study**

| **Name** | **Target** | **Sequence*** | **Reference** |
| --- | --- | --- | --- |
| *Primers used for cloning* | | | |
| OBM101 | LV LTR | Ggtacctttaagaccaatg | This study |
| OBM102 |  | gtcaagtagacctaggagagtggtcatccatcccatgcaggct |  |
| OBM103 |  | actctcctaggtctacttgacggaggtttgacagccgcctag |  |
| OBM104 |  | tctagatgctgctagagattttc |  |
| OBM156 | HIV *pol* | gtacatacagccaatggcagcaatttcaccag | [1] |
| OBM157 |  | tgccattggctgtatgtactgtttttactgg |  |
| OBM200 | HIV *tar* sequence | agagaactcccaggctcagatct | This study |
| OBM201 |  | gggagttctctggctaactagg |  |
| *Primers used for quantitative RT-PCR* | | | |
| OBM209 | human *xrcc5* gene (encoding Ku80) | ACCAAAGCGCCTGAGGAC | This study |
| OBM210 |  | CTCATGGTAAAGCCCACGTC |  |
| OBM257 | *hr-gfp* | aaacctggccctgtcttctt | This study |
| OBM258 |  | gaggaactgcttccttcacg |  |
| OBM24 | Human *β-actin* | AAATCTGGCACCACACCTTC | This study |
| R |  | TCACCGGAGTCCATCACGAT |  |
| OBM241 | Human *gapdh* | cgctctctgctcctcctgtt | [1] |
| OBM242 |  | ccatggtgtctgagcgatgt |  |
| OBM247 | ERV-FRD | AAAAAGGAAGAAGTTAACAGC | [2] |
| OBM248 |  | ATATAAAGACTTAGGTCCTGC |  |
| OBM249 | HML2-ERV-K-HP1 | GGCCATCAGAGTCTAAACCAC | From Leib-Mösch’s lab |
| OBM250 |  | CTGACTTTCTGGGGGTGGCCG |  |
| OBM251 | HML-5 | TGAAAGGCCAGCTTGCTG | [2] |
| OBM252 |  | CAATTAGGAAATTCTTTTCTAC |  |
| OBM253 | HML-1 | aaaatcagggaaatggagaatg | This study |
| OBM254 |  | cttgggcagctaaaggaatg |  |
| *Primers used for quantitative PCR* | | | |
| Alu1 | Human Alu | TCCCAGCTACTGGGGAGGCTGAGG | [3] |
| Alu2 |  | GCCTCCCAAAGTGCTGGGATTACAG |  |
| L-M667 | LV | ATGCCACGTAAGCGAAACTCTGGCTAACTAGGGAACCCACTG |  |
| Lambda-T | L-M667 | ATGCCACGTAAGCGAAACT |  |
| AA55M | LV | GCTAGAGATTTTCCACACTGACTAA |  |
| MH 531 |  | TGTGTGCCCGTCTGTTGTGT |  |
| MH 532-UNIV |  | ccgagtcctgcgtcgagaga | This study |
| HIV F | 2-LTR LV junction | GTGCCCGTCTGTTGTGTGACT | [3] |
| HIV R1 |  | ACTGGTACTAGCTTGTAGCACCATCCA |  |
| BGLOB sense | β-globin | CAACTTCATCCACGTTCACC | Control kit DNA; Roche Diagnostics, Basel, Switzerland |
| BGLOB antisense |  | ACACAACTGTGTTCACTAGC |  |
| *Probes used for quantitative PCR* | | | |
| LTR-FL | LV | CACAACAGACGGGCACACACTACTTGA (3’ fluoresceine) | [3] |
| LTR-FC |  | CACTCAAGGCAAGCTTTATTGAGGC (5’ LC red 640 dye and 3’-P) |  |
| MH-FL |  | CCCTCAGACCCTTTTAGTCAGTGTGGAA (3’ fluoresceine) |  |
| MH-LC |  | TCTCTAGCAGTGGCGCCCGAACAG (5’ LC red 640 dye and 3’-P) |  |
| HIV FL | 2-LTR LV junction | CCACACACAAGGCTACTTCCCTGA (3’ fluoresceine) |  |
| HIV LC |  | TGGCAGAACTACACACCAGGGC (5’ LC red 640 dye et 3’ phosphorylée) |  |
| *siRNA* (5’-3’sense sequence indicated) | | | |
| Ku80 2 | *xrcc5* mRNA encoding Ku80) | gcgaguaaccagcucauaadTdT | [4] |
| Ku80 1 |  | gagcuaauccucaagucggdTdT | [5] |
| Ku70 | *xrcc6* mRNA encoding Ku70) | GAUGCCCUUUACUGAAAAAdTdT | [6] |
| P53 | *tp53* mRNA | GUGAGCGCUUCGAGAUGUUdTdT | [7] |
| Ctl | Scramble sequence unrelated to human genome | GCCGGUAUGCCGGUUAAGUdTdT | [7] |

Abbreviations: HERV, human endogenous retrovirus; HIV, human immunodeficience virus; *hr-gfp*, *humanized* *Renilla* *green fluorescent protein*; LTR, long terminal repeat; LV, lentiviral; *pol*, *polymerase*; RT-Q-PCR, reverse transcription quantitative PCR; siRNA, small interfering RNA; *tar*, *trans*-activation response RNA element; *xrcc*, *x-ray cross-complementing group*.

**Supplementary references**

1. Manic G, Maurin-Marlin A, Galluzzi L, Subra F, Mouscadet JF, et al. (2012) 3' Self-Inactivating Long Terminal Repeat Inserts for the Modulation of Transgene Expression from Lentiviral Vectors. Hum Gene Ther Methods.

2. Seifarth W, Frank O, Zeilfelder U, Spiess B, Greenwood AD, et al. (2005) Comprehensive analysis of human endogenous retrovirus transcriptional activity in human tissues with a retrovirus-specific microarray. J Virol 79: 341-352.

3. Brussel A, Sonigo P (2003) Analysis of early human immunodeficiency virus type 1 DNA synthesis by use of a new sensitive assay for quantifying integrated provirus. J Virol 77: 10119-10124.

4. Nimura Y, Kawata T, Uzawa K, Okamura J, Liu C, et al. (2007) Silencing Ku80 using small interfering RNA enhanced radiation sensitivity in vitro and in vivo. Int J Oncol 30: 1477-1484.

5. Waninger S, Kuhen K, Hu X, Chatterton JE, Wong-Staal F, et al. (2004) Identification of cellular cofactors for human immunodeficiency virus replication via a ribozyme-based genomics approach. J Virol 78: 12829-12837.

6. Ayene IS, Ford LP, Koch CJ (2005) Ku protein targeting by Ku70 small interfering RNA enhances human cancer cell response to topoisomerase II inhibitor and gamma radiation. Mol Cancer Ther 4: 529-536.

7. Vitale I, Senovilla L, Jemaa M, Michaud M, Galluzzi L, et al. (2010) Multipolar mitosis of tetraploid cells: inhibition by p53 and dependency on Mos. Embo J 29: 1272-1284.
