# Supplementary material for: Impact of the Ku Complex on HIV-1 Expression and Latency
Source: PLoS One. 2013 Jul 29;8(7):e69691. doi: 10.1371/journal.pone.0069691 (PMC3726783; doi:10.1371/journal.pone.0069691)
Supplement: Text S1 — Supplementary materials and methods. (DOC) [file pone.0069691.s012.doc]

**Supplementary materials and methods**

***Plasmids and vector production***

The genetic organization of the LV vectors is presented in **Figure 1**. RRLSIN.cPPT.hPGK-gfp (shortened to “SIN-PGK”) is a SIN-LV vector containing the *enhanced gfp* transgene under the control of the human PGK internal promoter. SIN-PGK was generated as previously described [1]. pXCD3 is derived from pNL4.3 containing the HIV-1 genome (GenBank: AF324493.1) from which the *Nhe*I-*Bsa*BI region of the *env* gene was deleted (named HIV-1 NL4.3 *env^-^*) and the *Hpa*I-*Xho*I fragment (corresponding to the *nef* gene) was replaced by the compatible IRES-*humanized* *Renilla* *gfp* (*hr-gfp*) fragment from the pIRES-hrGFP 1a plasmid (Clontech Laboratories, Inc., Mountain View, CA, USA). pXCD3 IN(D116A) is an integrase-defective version of pXCD3 encoding a mutated (D116A) integrase. This mutation was introduced into pXCD3 by directed PCR mutagenesis produced using the QuikChange XL Site-Directed Mutagenesis Kit (Agilent Technologies, Inc., Santa Clara, CA, USA) according to manufacturer's indications and with OBM156/OBM157 primers (**Table S2**). HR’-CMV-gfp (shortened to “HR’-CMV”) is an LV vector containing the *gfp* gene under the control of the immediate early human CMV and U3 LTR promoters [2]. pXCD24 was derived from pHR’-CMV by deleting the *Cla*I-*Bam*HI fragment corresponding to the CMV promoter. The pXCD24 TAR(C37T) construct was generated by replacing the original *Kpn*I-*Xba*I 3’LTR fragment of pXCD24 by a TAR(C37T) mutated version. This latter was obtained after insertion of the original *Kpn*I-*Xba*I 3’LTR fragment into the pGem-T-easy vector (Promega Corporation, Madison, WI, USA) and directed mutagenesis (as mentioned above) with the OBM200/OBM201 primers (**Table S2**). The pXCD24 Sc^-216-196^ construct was obtained by replacing the putative KBS (CGGAGAGAGAAGTATTAGAGT) located between nucleotides -216 and -196 of the *Kpn*I-*Xba*I U3 3’LTR region of pXCD24 by a scrambled sequence (actctcctaggtctacttgac). This latter was generated after insertion of the *Kpn*I-*Xba*I 3’LTR region of pXCD24 into the pGem-T-easy vector (Promega) followed by fusion PCR. In this aim, two separate PCRs were performed on pXCD24 by using the OBM101/OBM102 primers (**Table S2**) for the first PCR, and the OBM103/OBM104 primers (**Table S2**) for the second PCR, following the manufacturer’s recommendations for *Taq* Platinium High Fidelity amplification reactions (Invitrogen, Life Technologies Co., Carlsbad, CA, USA). Then, 1/50 of each PCR product was mixed together to perform a third PCR without primers for the first 10 cycles (98°C for 10s/45°C for 10s/72°C for 30s), and with the OBM101/OBM104 primers (**Table S2**) for the last 20 amplification cycles (98°C for 10 s/60°C for 10 s/72°C for 30 s). The resulting PCR product was inserted into the pGem-T-easy vector (Promega) according to the manufacturer’s recommendations, thereby generating the *Kpn*I-*Xba*I 3’LTR mutated fragment containing the scrambled sequence. The *Kpn*I-*Xba*I 3’LTR mutated fragment containing the scrambled sequence in place of the putative KBS was reintroduced into pXCD24 to replace the original *Kpn*I-*Xba*I region, thereby generating the pXCD24 Sc^-216-196^ construct. All constructs were maintained and stored in the Sure2 *E. coli* host strain (Agilent Technologies). LV vectors were generated by calcium phosphate-mediated transfection as detailed in [1].

***Cell culture***

WT and *Ku80^+/-^* 70.32 human colorectal carcinoma epithelial HCT 116 cells (kindly provided by Dr E.A. Hendrickson) [3] were maintained in McCoy’s 5A medium. *TP53^-/-^* HCT 116 cells as well as *Ku80^+/-^* HCT 116 derivatives containing either a neomycin (Neo) or a hygromycin-Ku80 expression pcDNA3.1 plasmid (named A6 clone) (shortened as ‘pCtl’ and ‘pKu80’, respectively) were obtained from Horizon Discovery Ltd (Cambridge, UK) and maintained in McCoy’s 5A medium (under a 10 µg/ml hygromycin selection for pKu80 *Ku80^+/-^*HCT 116 cells). Human HEK 293T cells were maintained in Dulbecco’s modified Eagle medium. Human T CD4^+^ lymphoid lymphoma SUP-T1 cells [4] as well as human T CD4^+^ lymphoid leukemia Jurkat cells [5] and CEM-T4 cells (a naturally isolated subclone of the CEM line displaying high levels of surface CD4 expression; obtained from J.P. Jacobs through the AIDS Research and Reference Reagent Program, Division of AIDS, National Institute of Allergy and Infectious Diseases, National Institutes of Health) [6] were cultured in RPMI-1640. All media, purchased from Gibco (Life Technologies Co.), were supplemented with 10 % fetal bovine serum (PAA Laboratories GmbH, Pasching, Austria) and 1 % penicillin/streptomycin (100 units/mL). All cell lines used in this study were incubated at 37°C, under an atmosphere containing 5 % CO_2_.

***Transduction and transfection procedures***

The day before the transduction, 10^5^ cells/well were plated in 12-well plates. Transduction was performed by addition of the viral suspension to each cell line depending on its confluence estimated the day of transduction. Two days post-transduction, transgene expression was evaluated by cytofluorometry as described below. The m.o.i. was calculated according to the percentages of GFP^+^ cells observed during the linear phase of transduction. Alternatively, for reactivation experiments, cells were transduced and maintained in a 25 cm² flask. Upon one week, 10^5^ cells/well were plated in 12-well plates and treated one day later with 200 nM of TSA (Sigma-Aldrich, St Louis, MO, USA) or 10 ng/ml of TNFα (BD Biosciences, San Diego, CA, USA) for additional 24 h. Dimethyl sulfoxide (DMSO, Sigma-Aldrich) was used as negative control of TSA treatment. For experiments exploring the reactivation of transgene expression in a pure population of transduced cells, GFP^+^ cells were sorted two or three days after transduction at low m.o.i. (< 0.1). Cells were then cultured for six weeks and subjected every week to a 24 h TSA-treatment. For both reactivation experiments, transgene expression was finally determined by flow cytometry.

SiRNAs transfection was performed using custom-designed siRNAs duplexes (**Table S2**) targeting Ku80, Ku70, p53 encoding mRNA or a sequence unrelated to the human genome (Ctl) (all purchased from Eurogentec S.A., Seraing, Belgium) as previously described [7].

***Cytofluorometric analysis***

Transgene expression (*i.e.*, percentage and MFI of GFP^+^ cells) was estimated by flow cytometry using a FACSCalibur^TM^ cytofluorometer (BD Biosciences). Analyses were carried out with CellQuestTM software (BD Biosciences), upon gating on the events characterized by normal forward/side scatter parameters. To assess cell cycle distribution, cells were stained and analyzed as previously described [7].

***Immunoblotting***

Protein samples were prepared, separated, electrotransferred and immunoblotted using specific antibodies for Ku80, Ku70, p53 or β-actin [all from Santa Cruz Biotechnology, Inc., Santa Cruz, CA, USA; references (ref.) H-300, N3H10, DO-1, I-19, respectively], as previously described [7].

***Quantification of viral DNA species and of the antigen p24***

Two, 8, 24, 72 and 240 h post-transduction, cells were collected, centrifuged at 270 g and washed once with PBS. Thereafter, genomic DNA (gDNA) samples were prepared and Q-PCR was performed as previously described [1]. Primers and probes (**Table S2**) used for integrated vDNA nested PCR as well as LV total vDNA and 2-LTR amplification [performed on LightCycler (Roche Diagnostics, Basel, Switzerland)] are described in [8]. The cell equivalents in sample DNA as well as the copy number of total vDNA and integrated vDNA were calculated as previously described [1]. Alternatively, the copy number of 2-LTR circular vDNA forms was determined with reference to 10^2^ to 10^6^ copies of linearized pXCD3 plasmid or pGem-T-easy vector containing the XCD3 2-LTR sequence.

The quantification of HIV-1 p24 antigen in viral suspensions was performed using p24 HIV-1 antigen ELISA assays (PerkinElmer Life Sciences, Inc., Boston, MA, USA) following the manufacturer's recommendations.

***Chromatin immunoprecipitation assay***

ChIP assays were performed with the chromatin immunoprecipitation assay kit (Upstate Biotechnology, EMD Millipore Corporation, Billerica, MA, USA), as previously described [9] and with the following modifications. Briefly, 10^7^ infected cells at 0.3-0.4 m.o.i (that correspond to 10^4^ copies of total vDNA/μg) were treated with 1 % formaldehyde for 10 min at 37°C or 5 min at 4°C for the CEM-T4 or the SUP-T1 cell line, respectively. Cross-linked cells were harvested, washed with PBS, and lysed in sodium dodecyl sulfate (SDS) lysis buffer for 10 min at 4°C. Chromatin was sonicated (6 or 10 10-s pulses for the CEM-T4 or SUP-T1 cells, respectively) and centrifuged. Then, supernatants were diluted 10-fold with ChIP dilution buffer and precleared with salmon sperm DNA-protein A-agarose beads. Diluted extracts were kept (one tenth) for a direct Q-PCR (input DNA) or incubated with 1 μg/ml of specific antibodies for histone H3 or Ku80 (respectively from Upstate Biotechnology – ref. 06-755 or Santa Cruz Biotechnology – ref. H-300) followed by incubation with salmon sperm DNA-protein A-agarose beads (immunoprecipitated vDNA). Following extensive washing, bound vDNA fragments were eluted and the DNA was recovered by incubation in elution buffer and incubated with proteinase-K. The immunoprecipitated and input vDNA were then extracted and subjected to PCR quantification of LV total vDNA.

***RetroArrays experiments and analysis***

Total RNA from 4x10^6^ cells was extracted with the RNAeasy Mini kit (QIAGEN, GmbH, Hilden, Germany) and resuspended in 50µl of diethylpyrocarbonate (DEPC)-treated water (Gibco, from Life technologies). Genomic DNA contamination was removed by treating 30µg of all mRNA samples with 2U of Turbo DNase I (Ambion, from Life technologies) for 30min at 37°C, according to the manufacturer’s instructions. RNA was repurified on RNAeasy Mini kit columns (QIAGEN). The quantity and quality of the isolated RNA were determined by analysis on gel (1% TBE 0.5X) and with a Nanovue spectrophotometer (GE HealthCare, Piscataway, NJ, USA). The absence of genomic DNA was controlled by classic PCR on 50ng of RNA with *Taq* Platinium enzyme (Invitrogen, from Life technologies) and mixed oligonucleotide primers (MOP) [10] with the following amplification program : 94°C 2 min ; 30X (94°C 30S, 50°C 2 min, 72°C 2min); 72°C 7 min. Reverse transcription of mRNA, hybridization target synthesis and labeling MOP PCR as well as microarray preparation and hybridization and post-processing of retrovirus-specific microarrays were performed as previously described [10]. Two independent PCR labeling and microarrays experiments, with positive (mix of RNA) and negative (H_2_O) controls, were performed for each sample. RetroArray contains 57 human endogenous retroviruses (HERV) groups, each in triplicate. Images saved in the Bitmap file format were used to present the results by false color mapping. One result (representative of the triplicate) has been aligned for each microarray and each condition (**Figure S6**). Images saved in TIF file format were processed with ImaGene^TM^ 8.0 software tool package (BioDiscovery Inc., Los Angeles, US). Local background was subtracted, generating “Norm1” set of data. Data were then normalized to the total fluorescence of each microarray (data “Norm2”). Ratios of mean intensity of each locus in WT over *Ku80^+/-^* HCT 116 condition were calculated, as well as *p* values associated to a two-paired Student’s *t* test. Loci presenting a ratio superior or equal to 1.3 or inferior or equal to 0.7, and a *p* value inferior to 0.01 are presented in **Table S1**. In a second step, the distribution of log-intensity microarray spots for each microarray was used to normalize hybridization efficiencies of microarrays, bringing them all to an equivalent distribution (data “Norm3”). Given the relatively low number of spots per microarray, the following analyses were performed on “Norm1” and “Norm3” data. A statistical non-parametric analysis in Biometric Research Branch (BRB-ArrayTools, Dr. Richard Simon and BRB-ArrayTools Development Team) was conducted. The *p* value was set to the robust value of 0.01 in order to highlight loci differentially expressed. Candidate genes for differential expression between the conditions studied were selected from theses analyses (**Table S1**).

***Quantitative RT-PCR***

DNA-free RNA samples were prepared and RT-Q-PCR analyses were performed as previously described [1]. The *actin*, *glyceraldehyde-3-phosphate dehydrogenase* (*gapdh*), *hr-gfp*, or *xrcc5* genes or HERV loci were amplified by means of the corresponding primer pairs (**Table S2**), from 2µl (between 10 and 20ng) of complementary DNA in a final volume of 20µl of LightCycler DNA Master SYBR Green I mix, according to the manufacturer’s recommendations (Roche Diagnostics, Basel, Switzerland). For HERV loci expression analysis, the amplification was: 95°C 10 min; 40-45 x [95°C 1”; 50°C 5”; 72°C 10-12”]; 1 x [95°C 0”; 65°C 15”; 95°C 0” 0.1°C/sec). For other genes, the amplification program was the same with the following modifications: the hybridization temperature was 60°C, the elongation times were 6” (*gapdh*), 10” (*hr-gfp, actin*), or 15” (*xrcc5*). For Ku80 encoding mRNA quantification and HERV transcriptome analysis, fold-change values were calculated by comparative Ct analysis after normalizing for the quantity of *gapdh* or *actin* mRNA in the samples concerned [11]. For analysis of (HR-)GFP expression, copy numbers of *hr-gfp* and *gapdh* mRNAs were determined with reference to a standard curve obtained by amplification 10^2^ to 10^7^ copies of linearized cloned DNA with matching sequences, and used to calculate the normalized ratios.

***Statistical procedures***

Unless otherwise specified, experiments were performed in triplicates and independently repeated at least twice. Data were analyzed with Microsoft Excel (Microsoft Co., Redmond, WA, USA) and statistical significance was assessed by means of two-tailed Student’s *t* test (*, *p* < 0.05; **, *p* < 0.01)

**Supplementary references**

1. Manic G, Maurin-Marlin A, Galluzzi L, Subra F, Mouscadet JF, et al. (2012) 3' Self-Inactivating Long Terminal Repeat Inserts for the Modulation of Transgene Expression from Lentiviral Vectors. Hum Gene Ther Methods.

2. Miyoshi H, Takahashi M, Gage FH, Verma IM (1997) Stable and efficient gene transfer into the retina using an HIV-based lentiviral vector. Proc Natl Acad Sci U S A 94: 10319-10323.

3. Li G, Nelsen C, Hendrickson EA (2002) Ku86 is essential in human somatic cells. Proc Natl Acad Sci U S A 99: 832-837.

4. Smith SD, Shatsky M, Cohen PS, Warnke R, Link MP, et al. (1984) Monoclonal antibody and enzymatic profiles of human malignant T-lymphoid cells and derived cell lines. Cancer Res 44: 5657-5660.

5. Schneider U, Schwenk HU, Bornkamm G (1977) Characterization of EBV-genome negative "null" and "T" cell lines derived from children with acute lymphoblastic leukemia and leukemic transformed non-Hodgkin lymphoma. Int J Cancer 19: 621-626.

6. Foley GE, Lazarus H, Farber S, Uzman BG, Boone BA, et al. (1965) Continuous Culture of Human Lymphoblasts from Peripheral Blood of a Child with Acute Leukemia. Cancer 18: 522-529.

7. Vitale I, Senovilla L, Jemaa M, Michaud M, Galluzzi L, et al. (2010) Multipolar mitosis of tetraploid cells: inhibition by p53 and dependency on Mos. Embo J 29: 1272-1284.

8. Brussel A, Sonigo P (2003) Analysis of early human immunodeficiency virus type 1 DNA synthesis by use of a new sensitive assay for quantifying integrated provirus. J Virol 77: 10119-10124.

9. Thierry S, Marechal V, Rosenzwajg M, Sabbah M, Redeuilh G, et al. (2004) Cell cycle arrest in G2 induces human immunodeficiency virus type 1 transcriptional activation through histone acetylation and recruitment of CBP, NF-kappaB, and c-Jun to the long terminal repeat promoter. J Virol 78: 12198-12206.

10. Seifarth W, Frank O, Zeilfelder U, Spiess B, Greenwood AD, et al. (2005) Comprehensive analysis of human endogenous retrovirus transcriptional activity in human tissues with a retrovirus-specific microarray. J Virol 79: 341-352.

11. Pfaffl MW (2001) A new mathematical model for relative quantification in real-time RT-PCR. Nucleic Acids Res 29: e45.
